# Black phosphorus/calcium silicate-functionalized 3D hierarchical scaffolds: Coupling photothermal therapy with ion microenvironment-induced mineralization for bone repair

**DOI:** 10.1016/j.mtbio.2026.103446

**Published:** 2026-07-10

**Authors:** Xinyue Guan, Siyu Xu, Wenxin Meng, Quanli Li, Yuhui Liu, Chuan Wu, Zhongrong Chen, Guomin Wu

**Affiliations:** a College & Hospital of Stomatology, Anhui Medical University, Key Lab. of Oral Diseases Research of Anhui Province, Hefei, 230032, China; b School of Biomedical Engineering, Anhui Medical University, Hefei, 230023, China

**Keywords:** Black phosphorus, Calcium silicate, Bone defect, Biomineralization, Bone regeneration

## Abstract

The repair of large-scale bone defects remains a significant challenge in clinical orthopedics, often complicated by bacterial infection and limited self-healing capacity. Therefore, developing artificial scaffolds that can simultaneously prevent infection and orchestrate a regenerative microenvironment is critical for successful therapy. Herein, a hierarchical 3D scaffold (CaSiO_3_/PCL NFs + BP@PCL MSs) integrating calcium silicate (CaSiO_3_)-doped polycaprolactone (PCL) nanofibers and black phosphorus (BP)-encapsulated microspheres is engineered via combined electrospinning and electrospraying techniques. This design constructs a biomimetic architecture with dual photothermal-ionic regulation capabilities. Under near-infrared (NIR) irradiation, the scaffold exhibits superior photothermal conversion, which rapidly eradicates bacteria while accelerating the release of therapeutic ions to establish a pro-mineralization niche. Further in vitro and in vivo evaluations demonstrate that the scaffold exhibits excellent in situ mineralization capability while simultaneously promoting cell proliferation, migration, and osteogenic differentiation. Furthermore, the implanted scaffold effectively promotes vascularized bone regeneration at defect sites. This strategy, synergizing photothermal therapy with an optimized ionic microenvironment, provides a promising approach for bone tissue engineering applications.

## Highlights

• Hierarchical 3D scaffolds were engineered via electrospinning and electrospraying.
• NIR light triggers rapid antibacterial photothermal therapy and active ion release.
• Black phosphorus and calcium silicate synergize to drive in situ biomineralization.
• The photothermal-ionic platform effectively promotes vascularized bone regeneration.

## Graphical abstract

**Figure:**
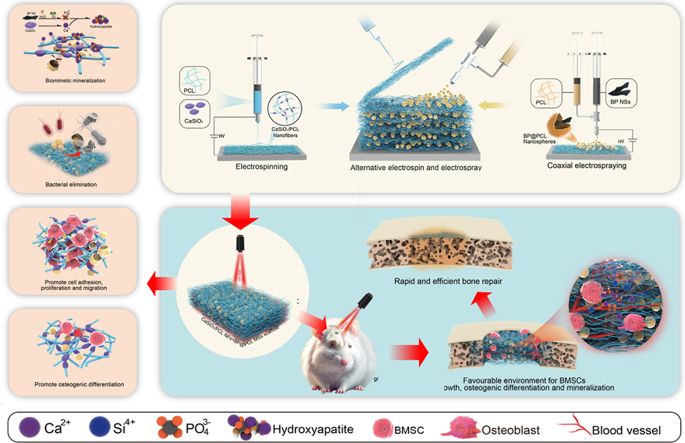

## 1 Introduction

The reconstruction of critical-sized bone defects remains a significant challenge in clinical orthopedics, with satisfactory solutions still elusive. [1]. While autogenous bone grafting persists as the gold standard due to its superior osteoinductivity and native microenvironment preservation [2], its widespread application is severely hindered by donor site morbidity, limited availability, and potential complications such as infection and hematoma [3]. Recently, the use of biodegradable bioactive scaffolds (such as hydrogels, nanofiber scaffolds, bioprinted scaffolds, bioactive ceramics, and gelatin sponges) to modulate the local microenvironment and promote tissue regeneration has emerged as a promising clinical strategy for repairing bone defects [[4], [5], [6], [7], [8]]. Among these, electrospun nanofiber scaffolds have garnered significant attention due to their ability to closely mimic the natural extracellular matrix (ECM) nanofibrous architecture, which provides a favorable microenvironment for cell adhesion, proliferation, and migration [9]. Numerous studies have demonstrated that these scaffolds can also promote stem cell differentiation and biomineralization, thereby facilitating bone regeneration [[10], [11], [12], [13]]. Furthermore, these nanofibers can function as versatile drug delivery systems, enabling efficient encapsulation and surface loading of bioactive agents [14,15]. Despite these advantages, conventional electrospinning generally produces densely packed two-dimensional (2D) membranes that cannot mimic the complex three-dimensional (3D) structure found in natural bone tissue, and their pores have limited connectivity, which severely restrict cell infiltration and nutrient transport [16]. Several strategies have been developed to transform 2D electrospun membranes into 3D nanofibrous scaffolds, including gas-foaming expansion, freeze-drying-assisted reconstruction, and multilayer stacking [[17], [18], [19]]. However, these methods primarily increase scaffold thickness and porosity, but they often compromise the nanofibrous topology. Gas-foaming may produce loose, unstable structures; freeze-drying alters the original fiber arrangement and adds extra steps; and multilayer stacking fails to improve interlayer pore connectivity, thus restricting cell infiltration throughout the scaffold. To overcome these limitations, integrating electrospraying with electrospinning offers a viable strategy to engineer porous scaffolds without complicating the fabrication process, thereby enhancing structural biomimicry.

In addition to structural design, constructing a favorable bioactive microenvironment via multi-physical cues is pivotal for enhanced osteogenesis [19,20]. Recent studies suggest that combining biophysical stimulation, such as photothermal therapy (PTT) induced by NIR light, with bioactive ions can improve regenerative outcomes compared with single-modality strategies [[21], [22], [23]]. While NIR-mediated PTT has shown promise in combating infection and stimulating stem cell differentiation [24], precise temperature control remains critical to avoid thermal necrosis of surrounding tissues [25,26]. In this context, two-dimensional black phosphorus nanosheets (BPNSs) [27] have emerged as a superior alternative to graphene oxide [21] or carbon nanotubes [28], demonstrating excellent photothermal conversion efficiency and biodegradability [[29], [30], [31]]. Notably, BPNSs can maintain optimal osteogenic temperatures under 808 nm NIR light irradiation [27] and degrades into phosphate-containing species [32]. According to previous studies [33], phosphorus rich materials can accelerate mineralization and promote bone regeneration by increasing the local concentration of phosphate anions and capturing calcium ions in the environment. Previous BP-based bone-regenerative systems have incorporated BPNSs into hydrogels, polymer scaffolds, or composite matrices and achieved promising results [[34], [35], [36]]. However, most of these designs directly disperse BPNSs within hydrated or polymeric networks, leaving them susceptible to water and oxygen penetration over time. Consequently, the long-term stability of their NIR-responsive photothermal performance and sustained phosphate release remains inadequately addressed. Moreover, photothermal stability is often evaluated only through short-term irradiation cycles [37], and systematic protection strategies against oxidation during prolonged implantation are limited. Therefore, encapsulating BPNSs in a protective carrier that delays oxidation while preserving NIR responsiveness and phosphate release represents a rational approach to enhance the long-term functionality of BP-based regenerative scaffolds.

Calcium silicate (CaSiO_3_) is a representative bioactive ceramic that has been widely investigated for bone regeneration [38]. Compared with conventional calcium phosphate ceramics, CaSiO_3_ exhibits superior biodegradability [39,40] while continuously releasing Ca^2+^ and SiO_4_^4-^-related ionic species that stimulate osteogenic differentiation and angiogenesis [41,42]. Furthermore, the abundant silicon hydroxyl groups (Si–OH) present on CaSiO_3_ surfaces provide favorable nucleation sites for hydroxyapatite (HA) deposition and mimic the mineralization-regulating functions of non-collagenous proteins during bone formation [[43], [44], [45]]. Importantly, the simultaneous release of Ca^2+^ from CaSiO_3_ and phosphate ions from BP degradation is expected to synergistically facilitate HA nucleation and in situ biomineralization, thereby creating a favorable microenvironment for bone regeneration [46]. Therefore, the combination of BP and CaSiO_3_ in a 3D nanofiber scaffold holds promise for the development of a multifunctional scaffold capable of facilitating efficient bone regeneration through the synergistic interaction between structural and biochemical signals.

**Fig. 1:**
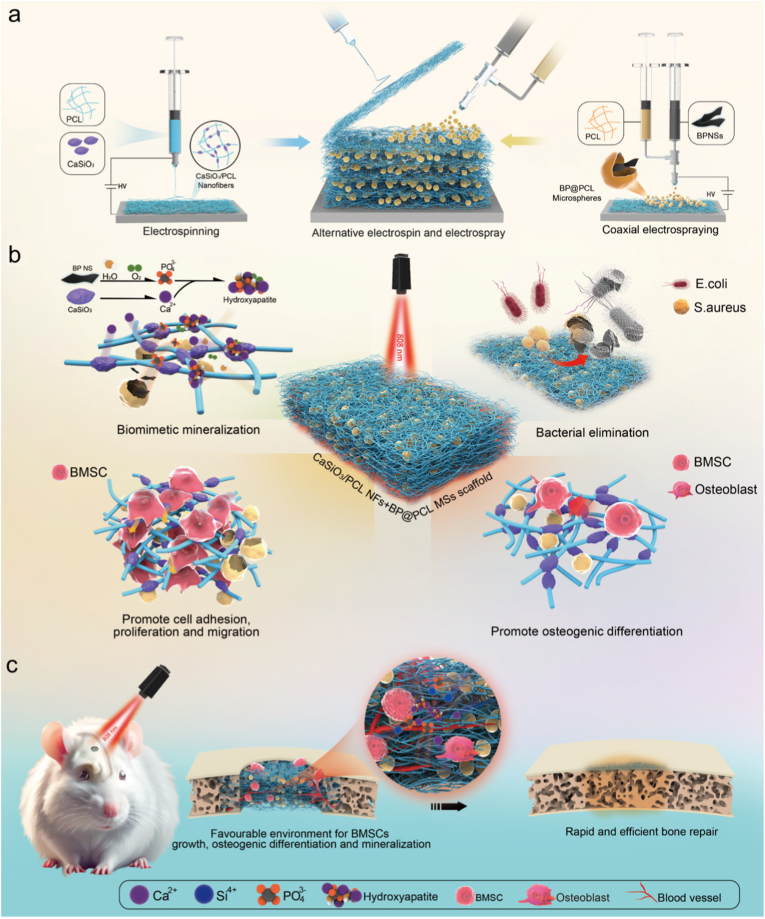
Schematic illustration of the multifunctional CaSiO_3_/PCL NFs + BP@PCL MSs 3D scaffold for bone defect repair. a) Fabrication process of the 3D scaffold. b) Multifunctional biological properties of the 3D scaffold under photothermal synergy: enhancing mineralization and antibacterial activity, promoting BMSC adhesion, proliferation, and migration, and inducing osteogenic differentiation. c) In vivo application for bone repair.

Herein, we developed a hierarchical 3D nanofibrous scaffold (CaSiO_3_/PCL NFs + BP@PCL MSs) designed to mimic both the 3D structural architecture and ion-regulated regenerative microenvironment of native bone. As illustrated in Fig. 1a, the scaffold was fabricated through an alternating strategy of electrospinning and coaxial electrospraying, in which CaSiO_3_-doped polycaprolactone (PCL) nanofibers were integrated with PCL microspheres encapsulated with BP. This architecture not only improves the spatial structure of conventional electrospun membranes but also protects BPNSs from rapid oxidation, thereby preserving their photothermal responsiveness under NIR irradiation (Fig. 1b). More importantly, the scaffold provides a dynamic ionic microenvironment composed of Ca^2+^, PO_4_^3−^, silicate-related species, which facilitates in situ hydroxyapatite mineralization and supports osteogenic cell responses. In vitro experiments demonstrated that this photothermal-ionic dual-regulation platform significantly enhances the adhesion, proliferation, and osteogenic differentiation of bone marrow mesenchymal stem cells (BMSCs). In vivo implantation further confirmed that the NIR-responsive scaffold effectively promotes vascularized bone regeneration in cranial defect models (Fig. 1c). Overall, this study proposes a facile strategy for constructing multifunctional 3D nanofibrous scaffolds and highlights their potential application as locally implantable regenerative scaffolds for non-load-bearing or low-load-bearing bone defects, particularly in craniofacial bone repair.

## 2 Methods

### 2.1 Preparation of BPNSs

Following the liquid stripping technique reported by Cheng et al. [17], BPNSs (MOA5228292, MossPhos, China) were prepared as follows: First, 10 mL of black phosphorus nanosheet dispersion was centrifuged at 4000 rpm for 15 min using a high-speed centrifuge, and the supernatant was collected. The supernatant was then centrifuged at 15,000 rpm for 10 min. The resulting BPNSs precipitate was dissolved in 1 mL of 1% Polyvinyl alcohol (PVA, Mw = 146,000∼186,000, P434375, Aladdin, China) solution.

### 2.2 Preparation of CaSiO_3_/PCL NFs + BP@ PCL MSs 3D scaffold

The CaSiO_3_/PCL NFs + BP@PCL MSs 3D nanofiber scaffold was prepared via alternating electrospinning and coaxial electrostatic spraying techniques. First, a 10% PCL (Mn = 60,000∼65,000, P169018, Aladdin, China) solution was prepared: 1 g PCL was dissolved in 10 g hexafluoroisopropanol (HFIP, H107501, Aladdin, China), containing 0.5% CaSiO_3_ (FM-Nano001, Feynman nano, China) solution as Solution A. A separate 4.25% PCL solution was prepared as Solution B. Subsequently, a 1% PVA solution was prepared at 80°C and stirred for 2.5 h, serving as Solution C. Collected BPNSs were dissolved in Solution C to prepare a 1% BPNSs solution as Solution D. Solution A was loaded into a 5-mL syringe mounted on a micro-syringe pump. The pump operated at 20 kV voltage, 1 mL/h flow rate, 26.5 cm collection distance, and 400 rpm rotation speed, with the electrospinning process lasting 1.5 h. Subsequently, solution B was introduced into the outer shaft of the coaxial needle at a flow rate of 1 mL/h, while solution D was introduced into the inner shaft at 0.4 mL/h. The coaxial needle voltage was set to 18 kV, with a collector distance of 26.5 cm and a collector rotation speed of 80 rpm. Under these conditions, electrospinning and coaxial electrostatic spraying were alternately cycled, ultimately completing 6 cycles of electrospinning and 5 cycles of coaxial electrostatic spraying. The resulting CaSiO_3_/PCL NFs + BP@PCL MSs 3D nanofiber scaffold was then dried in a fume hood to completely evaporate residual solvents.

### 2.3 Characterization

The morphology of BPNSs, fibers, microspheres, and scaffolds was observed using scanning electron microscopy (SEM, GeminiSEM 300, Zeiss, Germany), transmission electron microscopy (TEM, Talos L120C G2, Thermo Fisher Scientific, USA), and atomic force microscopy (AFM, NanoWizard 4XP, Bruker, Germany). ImageJ software (National Institute of Mental Health Research Service) was used to randomly measure the diameters of 100 BPNSs, 100 nanofibers, and 100 microspheres, respectively, and statistically analyze their diameter distribution data. The elemental composition of all scaffolds was analyzed using energy dispersive spectrometer (EDS) and X-ray photoelectron spectroscopy (XPS, Thermo Kalpha, Thermo Fisher Scientific, USA). Mechanical properties of 1 × 3 cm^2^ (length×width) scaffolds were tested using a universal testing machine equipped with a 10N sensor (travel speed 5 mm/min). Water contact angle (WCA) of all scaffolds was measured using a contact angle measurement system (OCA15EC, Dataphysics, Germany). After vacuum degassing at 37°C for 8 h, nitrogen adsorption-desorption isotherms were measured at 77 K using a specific surface area and pore size analyzer (ASAP 2460, Micromeritics, USA).

### 2.4 Evaluation of photothermal property

To evaluate the photothermal performance of the 3D scaffolds, testing was performed using an 808 nm near-infrared lamp and a handheld thermal imager. The 3D scaffolds were exposed to near-infrared excitation lamps at varying powers for 5 min, with temperatures recorded every 5 s using a handheld thermometer. All scaffolds were irradiated with a near-infrared light source at a wavelength of 808 nm and a power of 1 W cm^−2^. The CaSiO_3_/PCL NFs + BP@PCL MSs 3D scaffold underwent identical infrared irradiation conditions under varying thicknesses of porcine skin barrier protection, with temperature changes recorded. Finally, the CaSiO_3_/PCL NFs + BP@PCL MSs 3D scaffold underwent the aforementioned infrared irradiation followed by cooling over five wavelength cycles, with temperature changes concurrently recorded.

### 2.5 Release of Ca^2+^ and PO_4_^3−^ ions in vitro

The experiment was divided into non-NIR group and NIR group. 100 mg of the scaffold was immersed in 10 mL of deionized water and placed in a 37°C constant-temperature horizontal shaker. At various time points (0, 1, 2, 3, 5, 7, 14, and 18 days), 1 mL of the solution was sampled for testing, and the original volume was replenished with 1 mL of deionized water. The NIR group underwent the same procedure but was exposed to near-infrared light for 5 min daily. The release of Ca^2+^ and PO_4_^3−^ was measured using the Calcium Assay Kit (MAK477, Sigma-Aldrich, USA) and the Phosphate Assay Kit (MAK488, Sigma-Aldrich, USA), following standard operating procedures.

### 2.6 Evaluation of acellular mineralization ability

To evaluate the acellular mineralization ability of 3D scaffolds, a mineralization solution was prepared containing 1.67 mM CaCl_2_ (C4901, Sigma-Aldrich, USA), 9.5 mM Na_2_HPO_4_ (106,586, Sigma-Aldrich, USA), 150 mM NaCl (S9888, Sigma-Aldrich, USA) and 240 μg/mL Poly-aspartic acid (P-Asp) (P3418, Sigma-Aldrich, USA). After UV sterilization, all scaffolds were immersed in a sterile NaCl solution with pH 10.5 and a self-made sterile mineralization solution. At the appropriate time, all scaffolds were collected and SEM was used to observe the deposition of mineralization on its surface. Subsequently, XRD was used to identify mineralization, with a scanning crystallization range of 10-70°. And quantitatively analyze the content of mineralized deposits on the surface of all scaffolds. In order to quantitatively analyze the mineral content, all scaffolds were raised from room temperature to 600°C in the furnace. By comparing the weight changes before and after sintering, the calculation formula for mineral content is as follows:

$$\text{Mineral}\;\text{Content}=\frac{m1\;\left(\text{weight}\;\text{after}\;\text{sintering}\right)}{m0\;\left(\text{weight}\;\text{before}\;\text{sintering}\right)}*100\%$$

### 2.7 Evaluation of scaffold pH, and zeta potential

The scaffolds were immersed in PBS solution and placed in a horizontal shaker at 37°C. The NIR-treated samples were exposed to near-infrared light for 5 min daily. To assess the degradation status, The pH and zeta potential (Zetasizer Lab, Malvern Panalytical, UK) of the solution were measured and analyzed at, 1, 2, 3, 5, 7, 14, and 18 days.

### 2.8 Evaluation of antibacterial performance

The antibacterial performance of scaffolds was evaluated using Gram-positive *S. aureus* and Gram-negative *E. coli*. 30 μL of *S. aureus* suspension (1 × 10^5^ CFU/mL) was carefully dropped onto the surface of the scaffolds and moved to a 37°C incubator. After 4 h of cultivation, a NIR excitation lamp with a wavelength of 808 nm and a power of 1 W cm^−2^ was used to irradiate the scaffold for 10 min. Subsequently, the scaffolds were transferred to 1 mL of bacterial culture medium and cultured for 8 h. The antibacterial performance of the scaffolds was evaluated using agar plate counting and bacterial viability staining (L7012, Thermo Fisher Scientific, USA). E. Coli was detected using the same method as described above at a concentration of 1 ∗ 10^4^ CFU/mL.

### 2.9 Evaluation of the cellular biocompatibility of 3D scaffold

SD rat-derived BMSCs were used to evaluate cellular biocompatibility. BMSCs were cultured in a 37°C incubator with 5% CO_2_. All scaffolds were UV-sterilized, cut into 16 mm diameter discs, and fixed in 24-well plates. A cell suspension at 1 × 10^5^ cells/ml was uniformly seeded onto the scaffold surfaces of each group and cultured in a 37°C, 5% CO_2_ incubator. Scaffolds were irradiated with near-infrared light at 808 nm wavelength and 1 W cm^−2^ power for 10 min. After 3 days, BMSCs on the scaffolds were stained using a live/dead cell detection kit (04511, Sigma-Aldrich, USA). Fluorescent images were acquired via an upright fluorescence microscope (DM6 B, Leica, Germany), where green indicated live cells stained with calcein, and red indicated dead cells stained with propidium iodide. To further observe cell spreading morphology on the scaffolds, filamentous proteins were simultaneously stained. Specifically, cells were fixed with 4% paraformaldehyde, treated with 0.5% (v/v) Triton X-100, and labeled with FITC and DAPI to achieve dual staining of actin and nuclei. Finally, fluorescence images were acquired using a fluorescence microscope (DM6 B, Leica, Germany), and cell area measurements were performed with ImageJ software. To examine cell attachment to the scaffold, the scaffold was fixed with 4% paraformaldehyde and subjected to ethanol gradient dehydration. Images were then captured using a scanning electron microscope.

### 2.10 Assessment of cell migration capability on 3D scaffolds

All scaffolds were cut into circular shapes with a diameter of 16 mm and fixed in a 24 well plate after being sterilized by ultraviolet radiation. The polydimethylsiloxane (PDMS, Sylgard 184, Dow Corning, USA) strip was cut into 15.5 × 2 × 2 mm and fixed on the surface of the bracket. 1 mL of cell suspension with a concentration of 1 × 10^5^ cells/ml was uniformly inoculated on both sides of the strip. After overnight cultivation, remove the strips and allow the cells to migrate towards the middle. Irradiate with NIR excitation light with a wavelength of 808 nm and a power of 1 W cm^−2^ for 10 min. After 3 days, all scaffolds were removed and the myofilament proteins on the scaffolds were stained. Fluorescence images were obtained using a fluorescence microscope (DM6 B, Leica, Germany). To measure the cellular permeability of the 3D scaffold, cells were seeded onto the scaffold surface and cultured according to the aforementioned method. After five days, the cells' myofilaments were stained, and images were acquired using a laser confocal microscope (LSM880, Zeiss, Germany).

### 2.11 ALP staining and quantification

Remove the scaffolds after 7 days of induction. A portion was subjected to color development using the BCIP/NBT Alkaline Phosphatase Chromogenic Assay Kit (C3206, Beyotime, China), followed by observation and photography under a stereomicroscope. The remaining unstained scaffolds were lysed at room temperature for 30 min with lysis buffer, and the lysate was collected. Measure protein concentration in 30 μl samples using the BCA Protein Concentration Assay Kit (P0012, Beyotime, China). Determine ALP content in other solutions using the Alkaline Phosphatase Detection Kit, then calculate ALP activity and compile statistical data.

### 2.12 Alizarin Red S (ARS) Staining

Cell inoculation and culture were carried out using the same method as above. After 14 days and 21 days, all scaffolds were removed, fixed in 4% paraformaldehyde, and washed with deionized water. The scaffolds were stained with alizarin red for 30 min and the residual staining agent was removed with deionized water. The image was obtained using a stereoscopic microscope. The calcium nodules on each scaffold were dissolved by 500 μl of 100 mM cetylpyridinium bromide (CPB, H108698, Aladdin, China), and the enzyme-linked immunosorbent assay was used to read at a wavelength of 570 nm.

### 2.13 Immunofluorescence staining of ALP, BMP-2, col 1 and OCN

Remove the 7-day-induced scaffold, rinse three times with PBS, fix with 4% paraformaldehyde for 15 min, rinse three times with PBS, then disrupt membranes with 0.5% Triton X-100 solution for 15 min Followed by three rinses with PBS. Block with 5% goat serum at 37°C for 30 min. Incubate overnight at 4°C with primary antibody diluted 1:200 in PBS. Remove primary antibody, rinse three times with PBS. Incubate for 1 h in the dark with secondary antibody diluted 1:50 in PBS. Rinse three times with PBS. Add FITC-labeled filament green microfilaments diluted 1:200 in PBS and incubate in the dark for 30 min. Wash three times with PBS. Mount with DAPI-containing anti-fluorescence quenching mounting medium. Observe and photograph using a fluorescence microscope.

### 2.14 Quantitative real-time (qRT) polymerase chain reaction (PCR) assay

The expression of osteogenic genes ALP, OPN, Col I, BMP2, HSP47, and HSP70 was detected using qRT PCR method. All scaffolds were cut into circular shapes with a diameter of 35 mm and fixed in a 6 well plate after being sterilized by ultraviolet radiation. Inoculate and culture the cells according to the above method. After 7 days, all scaffolds were rinsed with PBS, and the total RNA of bone marrow mesenchymal stem cells cultured on the scaffolds was extracted using the Trizol method. Synthesize cDNA using a 1 μg RNA and reverse transcription first strand cDNA synthesis kit. In addition, the SYBR Premium Ex labeling kit and ABI 7500 sequencing detection system amplified cDNA through qRT PCR analysis.

### 2.15 Assessment of bone defect repair in vivo

Forty male SD rats with average weight of 200 g purchased from Animal Experiment Center of Anhui Medical University (Hefei, China) with the approval of the Ethics Committee (No. LLSC20210963) were used in this study. After anesthesia, bilateral cranial defect models were established by creating defects on both sides of the cranial suture using a 5-mm-diameter trephine bur. After sterilization by γ-ray irradiation, different scaffold groups were implanted into the two defect sites of the same rat. Subsequently, photothermal therapy (PTT) was performed in rats implanted with scaffolds containing BP@PCL MSs. The first NIR irradiation was conducted 4 h after surgery to avoid potential interference from surgical stress during the initial recovery of local tissues. Thereafter, NIR irradiation was performed once per week, with each irradiation session lasting 5 min. The interval between two irradiation sessions was 1 week, and a total of nine irradiation sessions were performed.

To evaluate the in vivo bone regeneration of implanted nanofiber scaffolds, a micro-CT scanner was used for detection, set at 70 kV, 141 μA, 1 mm aluminum filter, with a spatial resolution of 17.67 μm. Images were reconstructed by software. The software is used to calculate bone mineral density (BMD), bone volume fraction (BV/TV), and trabecular thickness (Tb.Th).

After scanning, all samples were immersed in decalcification solution for one month. The samples were embedded in paraffin and cut into 4 μm tissue samples for H&E, Masson, and immunohistochemical staining.

### 2.16 Statistical analysis

At least three samples were used in each group. All data are expressed as mean ± standard deviation. Statistical significance was assessed using one-way analysis of variance (ANOVA) with Tukey's post hoc test or two-tailed Student's t-test. ns: p > 0.05, ∗p < 0.05, ∗∗p < 0.01, ∗∗∗p < 0.001, ∗∗∗∗p < 0.0001.

## 3 Results and discussion

### 3.1 Preparation and characterization of electrospun nanofibers, coaxial electrospray nanospheres and BPNSs

**Fig. 2:**
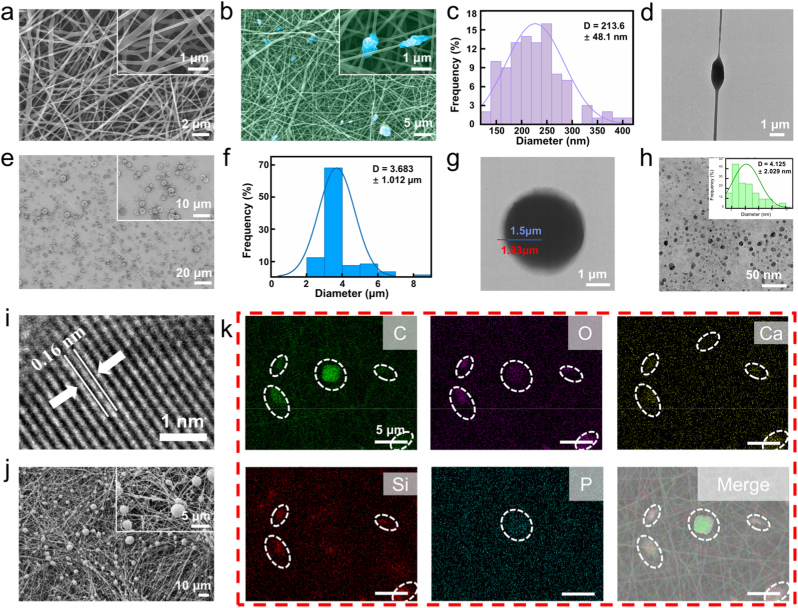
Characterizations of the electrospun nanofibers, coaxial electrospray nanospheres and BPNSs. a) SEM image of the electrospun PCL nanofibers. b) SEM images, c) fiber diameter distribution, and d) TEM image of CaSiO_3_-loaded CaSiO_3_/PCL nanofibers. e) SEM images, f) the diameter distribution and g) TEM image of the BP@PCL MSs. h) TEM image, the diameter distribution and i) the HR-TEM image of the BPNSs. j) SEM images of the distribution of PCL MSs on PCL NFs. k) EDS elemental mappings of the distribution of CaSiO_3_/PCL NFs on BP@PCL MSs.

PCL nanofibers (PCL NFs) and CaSiO_3_ loaded PCL nanofibers (CaSiO_3_/PCL NFs) were fabricated via electrospinning. Field-emission scanning electron microscopy (FE-SEM) showed a uniform fibrous morphology for both groups (Fig. 2a). Pseudocolored SEM images (Fig. 2b) showed the uniform distribution of CaSiO_3_ nodules (blue) along the PCL fibers. The average fiber diameter was 213.6 ± 48.1 nm (Fig. 2c), which falls within the range reported to support osteogenic responses [47]. The incorporation of CaSiO_3_ was further supported by TEM observation of embedded nodules (Fig. 2d) and EDS elemental mapping of Ca and Si (Fig. S1, Supporting Information). Subsequently, BP-encapsulated PCL microspheres (BP@PCL MSs) were prepared by coaxial electrospraying. SEM images exhibited spherical microspheres with an average diameter of 3.683 ± 1.012 μm (Fig. 2e and f). TEM observation supported the formation of a core-shell structure in BP@PCL MSs (Fig. 2g). High-resolution TEM of pure BPNSs showed an average diameter of 4.125 ± 2.029 nm (Fig. 2h) and lattice fringes with a d-spacing of 0.22 nm (Fig. 2i), consistent with typical BPNS structures [48]. The P elemental signal in the EDS mapping of the BP@PCL MSs further supported BP encapsulation (Fig. S2, Supporting Information). Finally, to construct the hierarchical composite scaffold, BP@PCL MSs were electrosprayed onto the CaSiO_3_/PCL NFs. SEM observation showed that the microspheres were uniformly anchored onto the nanofibrous network (Fig. 2j). Furthermore, EDS mapping of the composite scaffold displayed the co-localization of Ca, Si, and P elements, supporting the structural integration of BP@PCL MSs and CaSiO_3_/PCL NFs (Fig. 2k).

### 3.2 Characterization of CaSiO_3_/PCL NFs + BP@PCL MSs scaffold

**Fig. 3:**
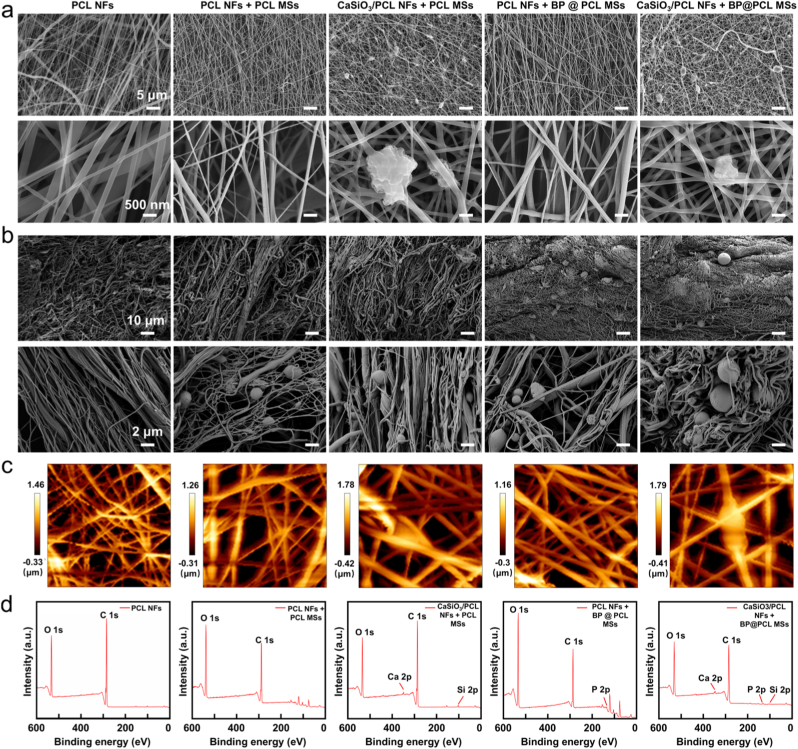
Characterizations of all scaffolds (PCL NFs, PCL NFs + PCL MSs, CaSiO_3_/PCL NFs + PCL MSs, PCL NFs + BP @ PCL MSs, CaSiO_3_/PCL NFs + BP@PCL MSs). a) SEM images of all scaffolds surfaces. b) SEM images of all scaffolds sections. c) AFM images of all scaffolds. d) XPS spectrum of all scaffolds.

A series of scaffolds, including pure PCL NFs and microsphere-incorporated composite scaffolds (PCL NFs + PCL MSs, CaSiO_3_/PCL NFs + PCL MSs, PCL NFs + BP@PCL MSs), and the final hierarchical platform (CaSiO_3_/PCL NFs + BP@PCL MSs), were successfully fabricated via combined electrospinning and coaxial electrospraying. Morphological evaluations via SEM and AFM showed that all scaffolds exhibited randomly oriented nanofibrous networks with relatively uniform fiber diameters (Fig. 3a–c). Cross-sectional SEM indicated that the assembled layers maintained structural continuity without obvious delamination (Fig. 3b). Notably, the incorporation of microspheres increased the scaffold thickness to approximately 239.5 μm, about 2.96 times that of the pristine nanofiber scaffolds (Fig. S3, Supporting Information). Furthermore, Brunauer–Emmett–Teller (BET) analysis demonstrated an increased specific surface area for the microsphere-incorporated scaffolds (Fig. S4, Supporting Information). This structural evolution is attributed to the microspheres acting as physical spacers between the nanofibrous layers, thereby increasing the internal pore size and overall volume. XPS analysis showed Ca 2p and Si 2p peaks at approximately 348.2 eV and 103.7 eV, respectively, in CaSiO_3_-containing scaffolds, while a P 2p peak at approximately 130.0 eV was detected in BP-containing groups (Fig. 3d).

Surface wettability, which is relevant to cellular behavior, was evaluated by dynamic WCA measurements. After 200 s, groups without CaSiO_3_ (PCL NFs, PCL NFs + PCL MSs, and PCL NFs + BP@PCL MSs) exhibited highly hydrophobic surfaces with WCAs ranging from 126° to 130°. In contrast, CaSiO_3_ incorporation decreased the WCAs to 5.55° ± 0.82° and 6.92° ± 1.25° for the CaSiO_3_/PCL NFs + PCL MSs and final composite scaffolds, respectively (Fig. S5, Supporting Information). This increase in hydrophilicity may favor cell adhesion and proliferation [9]. Finally, mechanical testing further showed that CaSiO_3_ incorporation increased the tensile strength from 1.69 MPa **for** pure PCL NFs to 3.53 MPa for the complete CaSiO_3_/PCL NFs + BP@PCL MSs scaffold (Fig. S6, Supporting Information), indicating improved mechanical stability for a flexible nanofibrous scaffold used in bone defect repair.

### 3.3 Photothermal properties of the CaSiO_3_/PCL NFs + BP@PCL MSs scaffold

**Fig. 4:**
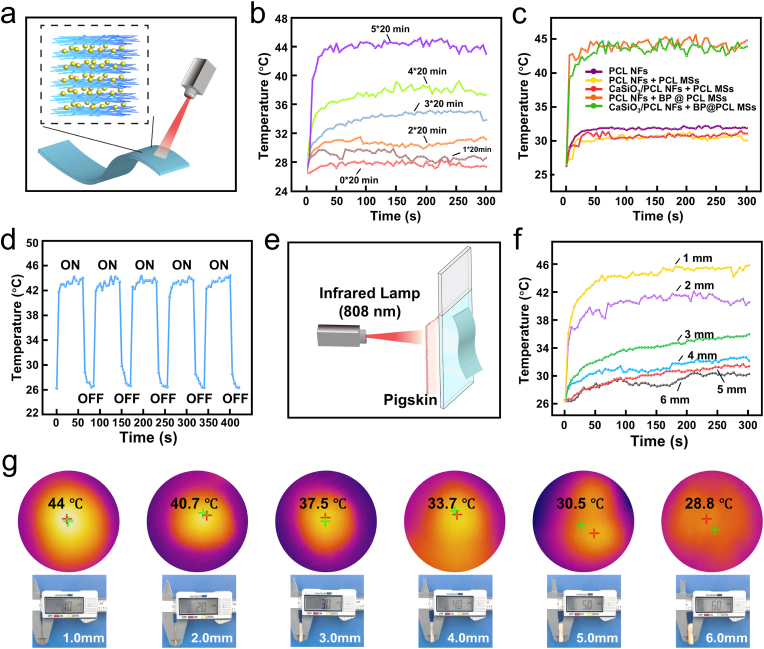
Photothermal performance of different scaffolds. a) Schematic diagram of the scaffolds exposed to NIR. b) Temperature in scaffolds with different BP@PCL MSs layers under NIR irradiation. c) The temperature of all scaffolds under NIR irradiation. d) Temperature changes of the CaSiO_3_/PCL NFs + BP@PCL MSs scaffold during 5 on/off cycles. e) Schematic diagram of pig skin blocking scaffold irradiation. f) The temperature of the scaffold irradiated by NIR under different thicknesses of pig skin barrier. g) Thermal images of the scaffold irradiated by NIR under different thicknesses of pig skin barrier.

Driven by the superior NIR absorption capacity of BPNSs, the composite scaffolds exhibit photothermal responsiveness. To evaluate this property, the scaffolds were exposed to an 808 nm NIR laser, and real-time surface temperatures were recorded (Fig. 4a). Under 1.0 W cm^−2^ irradiation, the terminal temperature of the scaffold exhibited a positive correlation with the number of BP electrospraying layers (Fig. 4b). Five electrospraying layers maintained the scaffold temperature within 42.5-45°C, a mild thermal range previously reported to support osteogenesis [26]. Further optimization showed that 1.0 W cm^−2^ rapidly increased the temperature to this range without excessive heating compared with 0.5 or 2.0 W cm^−2^ (Fig. S7, Supporting Information). BP-containing scaffolds reached the target temperature range, whereas BP-free control groups showed limited temperature changes (Fig. 4c). Furthermore, the CaSiO_3_/PCL NFs + BP@PCL MSs scaffold maintained similar peak temperatures over five consecutive laser on/off cycles (Fig. 4d), suggesting suitability for repeated NIR stimulation. To evaluate the feasibility of in vivo deep-tissue photothermal modulation, porcine skin tissues with thicknesses from 1 to 6 mm were placed above the scaffold to simulate biological barriers (Fig. 4e). As the tissue thickness increased, the peak temperature gradually attenuated from 45°C to 30°C, primarily due to light scattering and absorption by the tissue. Nevertheless, appreciable photothermal heating was still recorded even beneath 6 mm of tissue barrier (Fig. 4f and g), indicating that the scaffold could be activated through overlying soft tissue and may therefore be suitable for postoperative transdermal photothermal intervention in bone defect repair. This feature is particularly meaningful for clinical scenarios in which repeated local stimulation is required after implantation, as it avoids secondary surgical exposure while enabling external regulation of the defect microenvironment. In addition, we performed an in vivo photothermal heating experiment after scaffold implantation in rat cranial defects (Fig. S8, Supporting Information). The results showed that on day 18, the scaffold region could still be effectively heated under NIR irradiation. This further supports that encapsulation of BP within PCL microspheres can slow BP oxidation and help preserve its photothermal function over time Collectively, these results confirm that the CaSiO_3_/PCL NFs + BP@PCL MSs scaffold delivers a robust, precisely tunable, and highly reproducible photothermal performance tailored for controllable in vivo therapy. This externally regulated mild photothermal capability provides a practical foundation for subsequent antibacterial treatment, ion-release modulation, and osteogenic microenvironment construction after implantation, thereby supporting its potential application in bone defect repair.

### 3.4 Biomimetic mineralization performance of the CaSiO_3_/PCL NFs + BP@PCL MSs scaffold

The Ca^2+^ and PO_4_^3−^ release profiles confirmed that the CaSiO_3_/PCL NFs + BP@PCL MSs scaffold could establish a dynamically regulated pro-mineralization ionic microenvironment. Ca^2+^ was rapidly released at the early stage and then gradually entered a slow and sustained release phase. This behavior was mainly attributed to the rapid dissolution of CaSiO_3_ exposed on the surface of PCL nanofibers upon contact with water, followed by further ion release induced by water penetration into the interior of the PCL fibers. NIR irradiation slightly increased the cumulative release of Ca^2+^, indicating that mild photothermal stimulation promoted water penetration and ion diffusion (Fig. S9a, Supporting Information). In contrast, PO_4_^3−^ release exhibited a more obvious sustained-release and NIR-responsive pattern. In the absence of NIR irradiation, PO_4_^3−^ was slowly released from BP@PCL microspheres, suggesting that the PCL shell restricted the direct contact between BPNSs and the aqueous environment, thereby delaying the rapid degradation of BPNSs. Under NIR irradiation, the cumulative PO_4_^3−^ release increased, probably because mild photothermal stimulation promoted water diffusion into the microspheres and accelerated the gradual degradation of BPNS-loaded PCL microspheres (Fig. S9b, Supporting Information). Notably, PO_4_^3−^ maintained a sustained-release profile rather than burst release, further indicating that PCL encapsulation protected BPNSs and enabled controlled phosphate release.

**Fig. 5:**
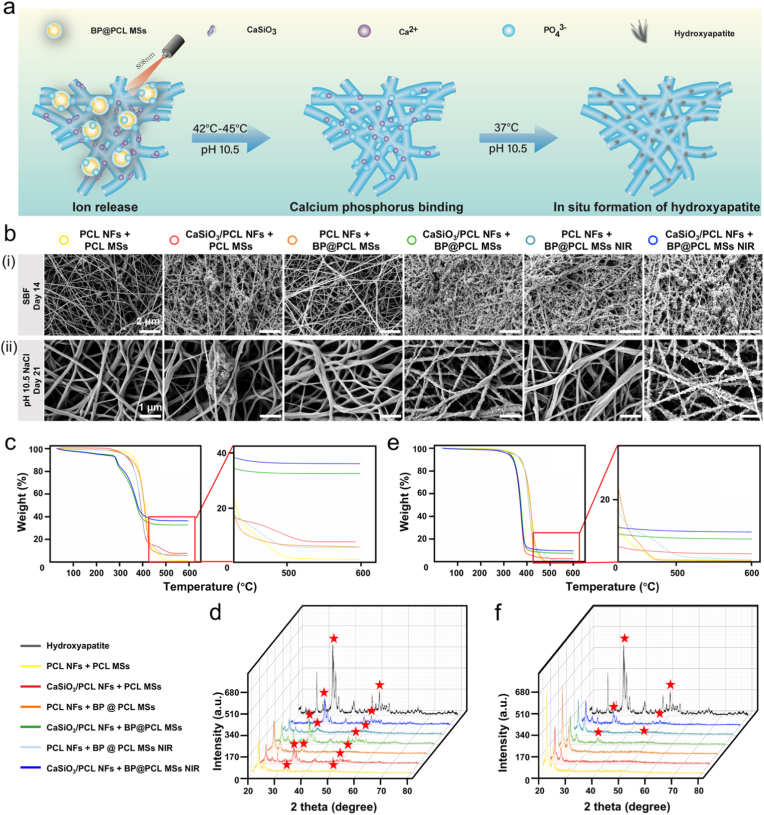
Acellular mineralization of different scaffolds. a) Schematic diagram of acellular mineralization. b) SEM images of different scaffolds soaked in (i) self-prepared mineralizing solution for 14 days, (ii) pH 10.5 NaCl for 21 days. The mineral of different scaffolds soaked in c) self-prepared mineralizing solution for 14 days, e) pH 10.5 NaCl for 21 days. XRD spectra of different scaffolds soaked in d) self-prepared mineralizing solution for 14 days. f) pH 10.5 NaCl for 21 days.

The sustained release of Ca^2+^ and the NIR-enhanced release of PO_4_^3−^ jointly increased the local calcium and phosphate ion concentrations, forming a supersaturated microenvironment favorable for calcium phosphate deposition, thereby promoting apatite nucleation and hydroxyapatite-like mineral formation. Meanwhile, CaSiO_3_-derived Si–OH groups and silicate-related species could serve as effective nucleation sites, further enhancing the biomimetic mineralization ability of the scaffold (Fig. 5a). SEM results showed that after incubation in mineralization solution for 14 days, scaffolds without CaSiO_3_ exhibited only sparse and uneven mineral deposition, whereas CaSiO_3_-containing scaffolds formed more extensive and dense mineralized layers (Fig. 5b(i)). Thermogravimetric (TG) analysis quantitative analysis further confirmed the increased mineral deposition after CaSiO_3_ incorporation (Fig. 5c).

To further verify the endogenous mineralization ability of the scaffold, the samples were immersed in NaCl solution without additional calcium or phosphate sources. The results showed that only the CaSiO_3_/PCL NFs + BP@PCL MSs scaffold formed relatively uniform mineral deposits, and the mineralization yield was further increased after NIR irradiation (Fig. 5b(ii), e). These findings indicate that NIR-mediated mild photothermal stimulation not only regulated ion release but also promoted BP degradation and PO_4_^3−^ liberation, thereby enhancing in situ calcium phosphate mineralization. XRD analysis further confirmed that the deposited minerals were characteristic HA phases (Fig. 5d and f). Taken together, the CaSiO_3_/PCL NFs + BP@PCL MSs scaffold provided Ca^2+^ and nucleation sites through CaSiO_3_, supplied controlled PO_4_^3−^ release through BP@PCL microspheres, and further enhanced ion release and mineralization under NIR stimulation, thereby constructing a photothermal-ionic synergistic microenvironment favorable for BMSC adhesion, osteogenic differentiation, and extracellular matrix mineralization.

In addition, zeta potential analysis of the immersion medium showed a gradual shift from −4 mV to −20 mV during 18 days of incubation, suggesting the progressive release of negatively charged degradation products and the evolution of the ionic microenvironment during scaffold degradation (Fig. S10a, Supporting Information). The pH of the medium remained nearly stable, indicating that the degradation process did not induce significant microenvironmental disturbance (Fig. S10b, Supporting Information). Furthermore, XPS analysis after 14 days confirmed the decrease of Ca-related signals and the presence of phosphorus species, supporting the dissolution of CaSiO_3_ and the retention/transformation of BP-derived components (Fig. S11, Supporting Information). These results collectively indicate a stable degradation-associated physicochemical evolution without disturbing the ion-driven mineralization process.

### 3.5 In vitro antibacterial activity of the CaSiO_3_/PCL NFs + BP@PCL MSs scaffold

**Fig. 6:**
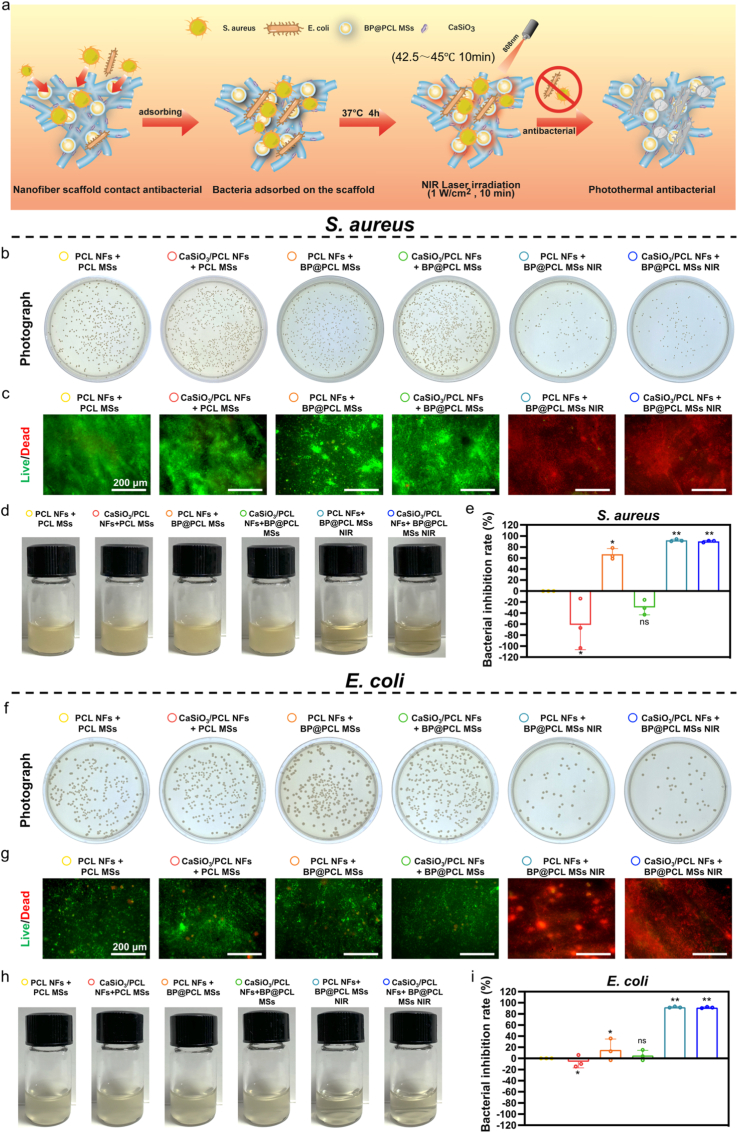
In vitro antibacterial performance of the scaffolds. a) Schematic illustration of the in vitro antibacterial experiment. b-e) Antibacterial evaluation against *S. aureus*: b) representative photographs of bacterial colonies on agar plates, c) fluorescence images of Live/Dead staining assays (green: live bacteria; red: dead bacteria), d) optical images showing the turbidity of bacterial suspensions, and e) statistical analysis of the bacterial inhibition rates. f-i) Antibacterial evaluation against *E. coli*: f) representative photographs of bacterial colonies, g) fluorescence images of Live/Dead staining, h) optical images of bacterial suspensions, and i) statistical analysis of the bacterial inhibition rates. ns: p > 0.05, ∗p < 0.05, ∗∗p < 0.01, compared with PCL NFs + PCL MSs group. (For interpretation of the references to color in this figure legend, the reader is referred to the Web version of this article.)

To evaluate antibacterial efficacy, *Staphylococcus aureus* (*S. aureus*) and *Escherichia coli* (*E. coli*) were seeded onto the samples and cultured with or without NIR irradiation (Fig. 6a). The results revealed that in the absence of NIR irradiation, only the PCL NFs + BP@PCL MSs scaffold exhibited moderate intrinsic antibacterial activity, yielding inhibition rates of 66.9 ± 10% against *S. aureus* (Fig. 6b–d, e) and 15.3 ± 19.6% against *E. coli* (Fig. 6f–h, i). These results suggest that the moderate antibacterial effect observed in the BP-containing scaffold was mainly derived from the intrinsic antibacterial activity of BPNSs [49]. Under the same conditions, neither the PCL NFs + PCL MSs scaffold nor the CaSiO_3_/PCL NFs + PCL MSs scaffold showed obvious antibacterial activity. In contrast, the CaSiO_3_-containing scaffold even appeared to promote bacterial adhesion, which may be attributed to the bioactive and hydrophilic surface of CaSiO_3_. However, the introduction of NIR irradiation (808 nm, 1.0 W cm^−2^ for 10 min) overcame this limitation via its excellent photothermal effect. Upon NIR activation, the antibacterial activity of BP-containing scaffolds increased markedly. The CaSiO_3_/PCL NFs + BP@PCL MSs scaffold achieved exceptional inhibition rates of 90.2 ± 1.6% against *S. aureus* and 91.4 ± 1.0% against *E. coli*. These outcomes showed no significant statistical difference compared to the PCL NFs + BP@PCL MSs group (92.0 ± 2.0% and 90.2 ± 1.6%, respectively). These quantitative findings were further corroborated by live/dead fluorescence staining assays, where viable bacteria emit green fluorescence and membrane-compromised dead bacteria exhibit red fluorescence. As depicted in Fig. 6c and g, the NIR-irradiated, BP-containing scaffolds exhibited predominantly red fluorescence, which stands in stark contrast to the dense green fluorescence observed in the non-irradiated control groups. These findings indicate that the scaffold combines CaSiO_3_-associated osteogenic bioactivity with externally activatable BP-mediated antibacterial function. Such an on-demand antibacterial strategy may be useful for postoperative management of infected or infection-susceptible bone defects, while avoiding reliance solely on systemic antibiotics or additional invasive procedures.

### 3.6 Biocompatibility of the CaSiO_3_/PCL NFs + BP@PCL MSs scaffold

**Fig. 7:**
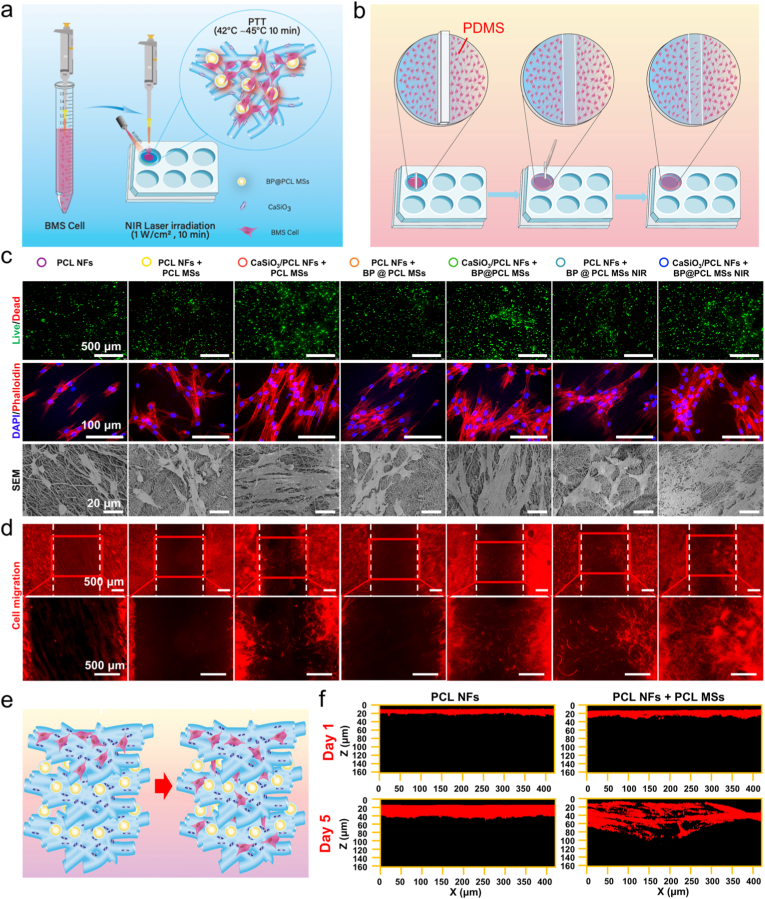
In vitro biocompatibility evaluation of BMSCs. a, b) Schematic illustrations depicting enhanced a) cell adhesion, proliferation, and spreading, and b) cell migration on the CaSiO_3_/PCL NFs + BP@PCL MSs scaffold combined with mild PTT. c) Representative Live/Dead staining, cytoskeleton staining, and SEM morphology images of BMSCs cultured on various scaffolds for 3 days. d) Fluorescent images of F-actin staining showing BMSC migration over different scaffold surfaces after 3 days. e) Schematic illustrating how mild PTT enhances cell infiltration into the interior of CaSiO_3_/PCL NFs + BP@PCL MSs 3D scaffolds. f) Fluorescent images of F-actin staining showing BMSC infiltration into 3D scaffolds after 5 days of mild PTT treatment.

Excellent biocompatibility is a fundamental prerequisite for biomedical implants. To systematically evaluate the synergistic effects of the scaffold architecture and NIR-induced mild hyperthermia on cell viability, BMSCs were seeded onto the scaffolds and subjected to periodic NIR irradiation (Fig. 7a). The macroscopic temperature was precisely maintained at ≈42°C, a thermal window previously validated to optimize osteogenesis [29]. Live/dead staining assays revealed exceptional cell viability across all groups with negligible cytotoxicity (Fig. 7c). Quantitative analysis (Fig. S12, Supporting Information) demonstrated that BMSC proliferation on the hierarchical 3D scaffolds was significantly higher than on conventional 2D PCL NFs, owing to the enlarged interconnected pores that facilitate efficient nutrient diffusion. Notably, the CaSiO_3_/PCL NFs + BP@PCL MSs scaffold supported the highest cell density. This result may be associated with CaSiO_3_-enhanced hydrophilicity, the release of bioactive ions, and mild PTT stimulation [27]. Cytoskeletal staining (F-actin/DAPI) confirmed that BMSCs on the composite scaffolds exhibited a well-spread, elongated spindle morphology with highly organized cytoskeletal networks (Fig. 7c). SEM observations further showed that the 3D micro/nanofibrous architecture provided surface area and topographical features for cell anchorage and extension. Furthermore, scaffolds incorporating CaSiO_3_ exhibited superior cell adhesion compared to pure PCL counterparts, a benefit derived from the enhanced hydrophilicity and surface bioactivity. Crucially, the application of NIR irradiation further augmented these cellular adhesive behaviors. Rapid BMSC migration to the defect site is crucial for early-stage bone healing. A physical barrier assay utilizing PDMS strips was employed to evaluate directional cell migration (Fig. 7b). Fluorescence imaging (Fig. 7d) revealed that CaSiO_3_-containing scaffolds recruited more migrating cells than CaSiO_3_-free groups, which may be related to the chemotactic effects of silicate [50]. Moreover, NIR-irradiated groups exhibited substantially accelerated cell migration compared to non-irradiated controls, confirming that mild thermal stimulation dynamically boosts cellular motility [13]. Finally, to regenerate mature bone, scaffolds must support deep cellular infiltration to reconstruct the native 3D architecture. Confocal laser scanning microscopy (CLSM) after 5 days of culture (Fig. 7e) demonstrated that the 3D microsphere-integrated scaffolds allowed extensive downward cellular penetration, whereas cells on conventional 2D PCL NFs remained confined to the superficial layer (Fig. 7f). This deep in vitro cellular infiltration is directly facilitated by the hierarchical porosity and expanded internal voids created by the microsphere spacers. Collectively, these results robustly demonstrate that the engineered composite scaffold, coupled with mild NIR photothermal stimulation, not only guarantees excellent biocompatibility but also provides an optimal 3D bioactive microenvironment that synergistically drives BMSC adhesion, proliferation, migration, and deep tissue infiltration.

### 3.7 In vitro osteogenic differentiation of the CaSiO_3_/PCL NFs + BP@PCL MSs scaffold

**Fig. 8:**
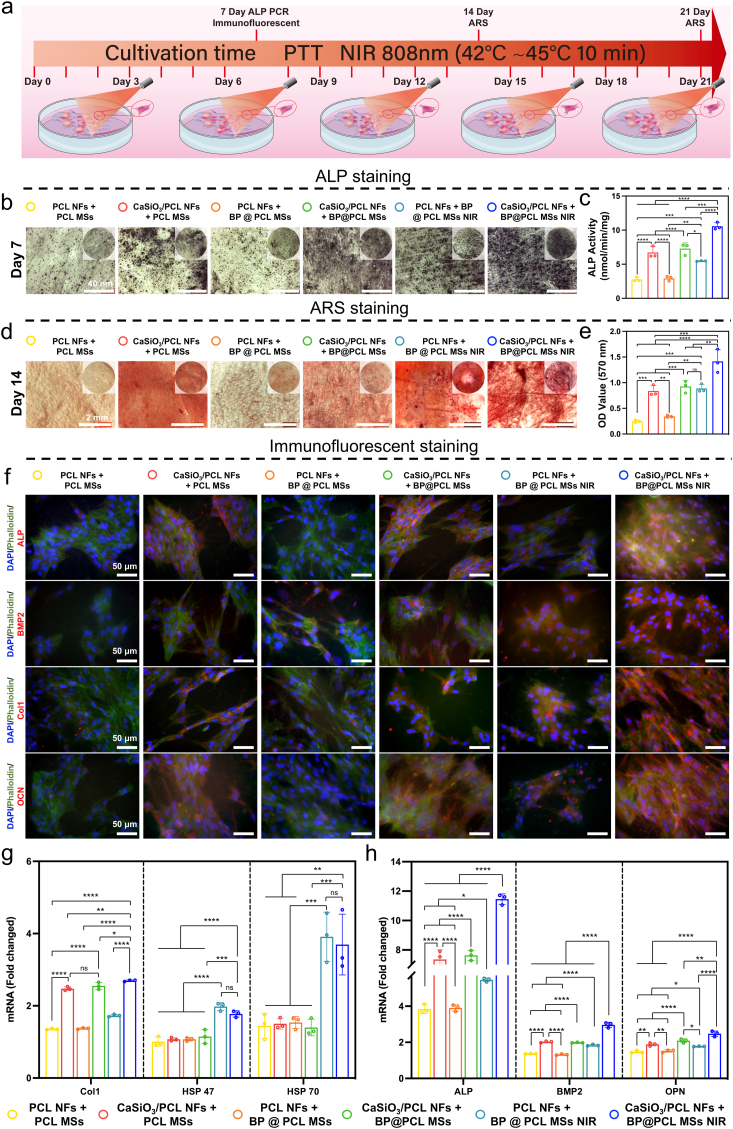
In vitro osteogenic differentiation induction on scaffolds a) Schematic of in vitro osteogenic differentiation induction on CaSiO_3_/PCL NFs + BP@PCL MSs scaffolds. b),c) Alkaline phosphatase staining and quantification of osteogenic-induced cells on the scaffold surface after 7 days. d),e) Alizarin red staining and semi-quantification of osteogenic-induced cells on the scaffold surface after 14 days. f) Immunofluorescence staining of osteogenic-induced cells on the scaffold surface after 7 days. g1), g2) Relative mRNA levels of osteogenic genes (Col-1, HSP47, HSP70, ALP, BMP-2, and OPN) in cells on the scaffold surface, determined by qRT-PCR. ns: p > 0.05, ∗p < 0.05, ∗∗p < 0.01, ∗∗∗p < 0.001, ∗∗∗∗p < 0.0001. (For interpretation of the references to color in this figure legend, the reader is referred to the Web version of this article.)

To evaluate osteogenic differentiation, BMSCs were cultured on the scaffolds and subjected to periodic NIR irradiation to maintain a mild hyperthermic environment (≈42 ± 0.5°C) (Fig. 8a). Alkaline phosphatase (ALP) is widely recognized as a crucial early-stage biomarker of osteogenesis that drives the initiation of extracellular matrix (ECM) mineralization [51,52]. After 7 days of culture, ALP staining and quantitative analysis (Fig. 8b and c) showed that scaffolds incorporating CaSiO_3_ exhibited significantly upregulated ALP expression compared to CaSiO_3_-free controls, supporting the osteogenic contribution of Ca^2+^ and silicate-related species. Remarkably, the CaSiO_3_/PCL NFs + BP@PCL MSs scaffold under NIR irradiation demonstrated the highest ALP activity with the other tested groups, suggesting a combined effect of mild PTT and thermally regulated release of PO_4_^3−^, Ca^2+^, and silicate-related species [27]. Furthermore, mature ECM calcium deposition—a definitive marker of late-stage osteogenic differentiation—was evaluated via Alizarin Red S (ARS) staining. On days 14 and 21 (Fig. 8d, e, and Fig. S13, Supporting Information), both the morphological staining and quantitative extraction assays corroborated the ALP trends. The NIR-irradiated CaSiO_3_/PCL NFs + BP@PCL MSs scaffold exhibited the most extensive and dense calcium nodule formation. The intermediate groups featuring either ionic cues (CaSiO_3_) or photothermal cues (BP + NIR) displayed moderate mineralization, whereas the baseline pristine scaffolds exhibited minimal calcium deposition. Concurrently, the translational expression of key osteogenic marker proteins—ALP, bone morphogenetic protein-2 (BMP-2), type I collagen (Col-1), and osteocalcin (OCN) [53]—was assessed via immunofluorescence staining. Imaging performed on day 7 (Fig. 8f) showed similar trends. BP-containing scaffolds also showed increased BMP-2 expression compared with pure PCL constructs, which may be related to BP-derived phosphate species and mild NIR-PTT [13]. To elucidate the underlying molecular mechanisms driving this synergistic osteogenesis, the transcriptional profiles of the BMSCs were quantified via qRT-PCR. Previous studies established that appropriate mild thermal stimulation upregulates heat shock proteins (HSPs) [54], which subsequently participate in bone metabolism and Col-1 biosynthesis [25]. Specifically, HSP47 is critical for cartilage and endochondral osteogenesis [13], while HSP70 enhances ALP activity and mesenchymal stem cell differentiation [26]. The results (Fig. 8g) showed that the gene expressions of HSP47 and HSP70 were significantly upregulated only in the NIR-irradiated groups (PCL NFs + BP@PCL MSs and CaSiO_3_/PCL NFs + BP@PCL MSs), with no significant statistical difference between the two groups. This confirms that controlled NIR-PTT can activate HSP-related responses in BP-containing scaffolds, and this effect is not primarily dependent on the incorporation of CaSiO_3_. Therefore, the upregulation of HSP47 and HSP70 further supports the role of NIR-triggered photothermal stimulation in promoting the HSP-associated osteogenic regulatory pathway. To further evaluate the downstream genetic effects, the expression levels of representative osteogenic genes, including ALP, BMP2, and OPN, were examined. Consistent with the protein-level findings, the NIR-irradiated CaSiO_3_/PCL NFs + BP@PCL MSs composite induced the most significant upregulation of all tested osteogenic genes (Fig. 8h). In summary, these findings suggest that the synergistic integration of an osteoinductive multi-ionic microenvironment, involving Ca^2+^, PO_4_^3−^, and silicate-related species, with controlled mild PTT within a 3D scaffold framework effectively promote osteogenic differentiation and support bone regeneration.

### 3.8 In vivo bone regeneration of the CaSiO_3_/PCL NFs + BP@PCL MSs scaffold

**Fig. 9:**
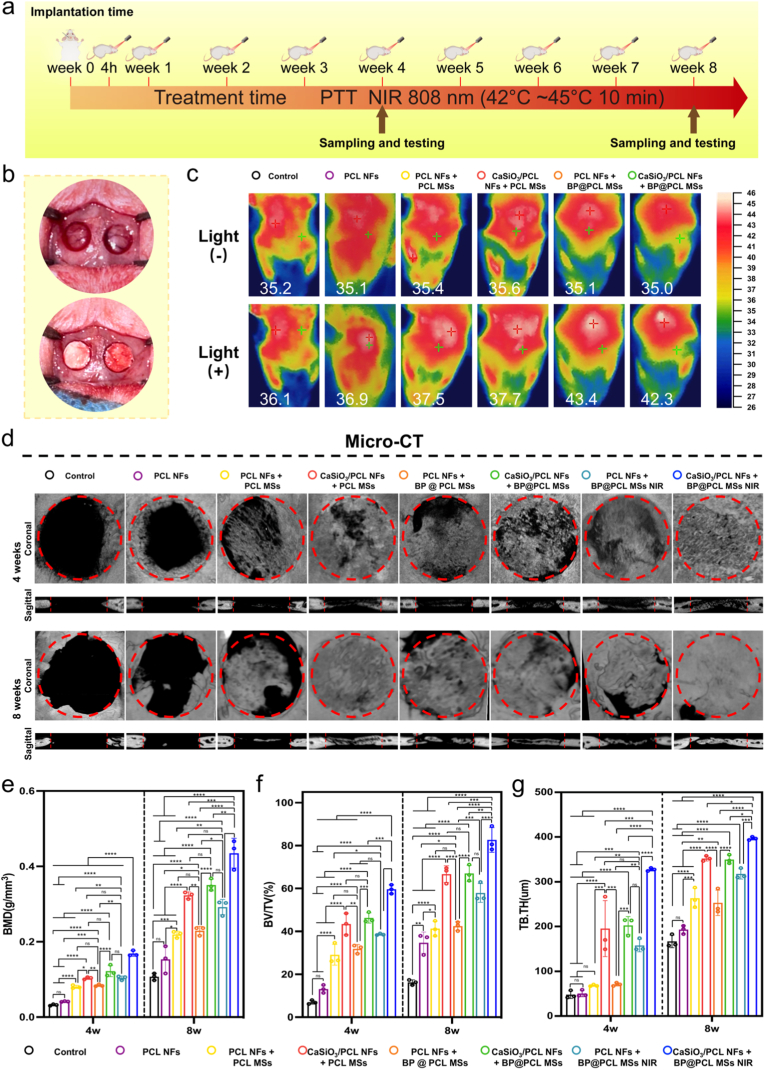
In vivo evaluation of cranial defect repair and photothermal therapy. a) Schematic illustration of the cranial defect modeling and the scaffold-mediated in vivo PTT treatment strategy. b) Surgical photographs showing the establishment of the critical-sized cranial defect and scaffold implantation. c) Infrared thermal images monitoring the in vivo temperature elevation of the scaffolds. d) Representative coronal and sagittal Micro-CT images of the defect area at 4 and 8 weeks post-treatment. e-g) Quantitative Micro-CT morphometric analysis of the regenerated bone within the defect area: e) BMD, f) BV/TV, and g) Tb.Th. ns: p > 0.05, ∗p < 0.05, ∗∗p < 0.01, ∗∗∗p < 0.001, ∗∗∗∗p < 0.0001.

To comprehensively evaluate the in vivo bone regenerative efficacy of the engineered scaffolds, a rat critical-sized bone defect model was established. After implantation, localized mild photothermal treatment was periodically applied under NIR irradiation, maintaining the defect-site temperature within a biologically safe and effective mild hyperthermia range of approximately 42.5–45°C (Fig. 9a and b). In vivo thermographic monitoring showed that the BP-incorporated scaffolds (PCL NFs + BP@PCL MSs and CaSiO_3_/PCL NFs + BP@PCL MSs) induced a localized temperature increase under NIR irradiation. Conversely, the control and BP-free groups showed only limited thermal responses under the same irradiation conditions (Fig. 9c).

Bone regeneration was longitudinally monitored using micro-computed tomography (micro-CT) at 4 and 8 weeks post-operation (Fig. 9d). At 4 weeks, the blank control and 2D PCL NFs groups exhibited limited new bone formation, mainly at the defect margins. In stark contrast, all 3D microsphere-integrated scaffolds induced extensive new bone formation across the defect area. By comparison, the 3D microsphere-integrated scaffolds promoted more extensive new bone formation within the defect area, suggesting that the hierarchical porous 3D architecture could serve as a structural bridge to support cell adhesion, migration, and tissue ingrowth [17]. By week 8, the control and pure PCL NFs groups still obvious unhealed defect areas, whereas the 3D composite scaffolds showed progressive and bone consolidation. Among them, the NIR-irradiated CaSiO_3_/PCL NFs + BP@PCL MSs scaffold exhibited the most evident bony bridging and defect repair.

Quantitative morphometric analyses, including BMD, BV/TV, and Tb.Th, were consistent with the reconstructed micro-CT observations (Fig. 9e–g). The 3D microsphere-containing groups showed higher values than the control and 2D PCL NFs groups at both time points. Furthermore, the incorporation of CaSiO_3_ further enhanced bone regeneration compared with the CaSiO_3_-free counterparts, supporting the osteogenic contribution of Ca^2+^ and silicate-related species released from the scaffold. In addition, the NIR-irradiated PCL NFs + BP@PCL MSs scaffold exhibited enhanced bone repair at 8 weeks compared to its non-irradiated may contribute to bone repair by providing localized thermal regulation and promoting BP-derived PO_4_^3−^ release for osteogenic mineralization.

Overall, the CaSiO_3_/PCL NFs + BP@PCL MSs + NIR group achieved the most favorable bone regeneration among all groups at both time points. These results suggest that the combination of a hierarchical 3D scaffold architecture, Ca/P/Si-related ionic microenvironment, and controllable mild photothermal stimulation can synergistically support in situ bone regeneration.

**Fig. 10:**
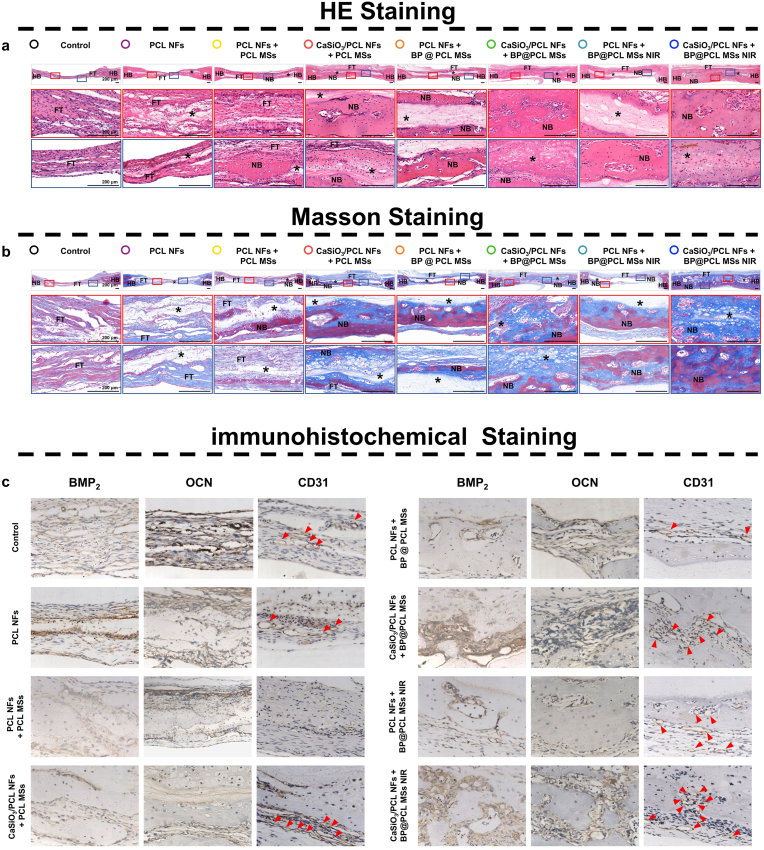
Histological and immunohistochemical assessment of cranial defects at 4 weeks post-implantation. Representative images of a) HE staining, b) Masson staining, and c) immunohistochemical staining for BMP2, OCN, and Cd31. The bottom panels present high-magnification views of the regions outlined in red and blue boxes. FT: fibrous tissue; NB: newly formed bone; ∗: scaffold material; red arrows: blood vessels. (For interpretation of the references to color in this figure legend, the reader is referred to the Web version of this article.)

To further evaluate the quality and maturity of the regenerated bone at the histological level, hematoxylin and eosin (H&E) and Masson's trichrome staining were performed on the harvested tissues. Histological observations at 4 (Fig. 10a and b) and 8 weeks (Fig. S14 and 15, Supporting Information) showed progressive degradation of the implanted scaffolds across all experimental groups, without obvious necrosis or severe inflammatory responses. These results indicate good in vivo biocompatibility and suitable biodegradability of the scaffolds, allowing them to provide temporary structural support while permitting continuous tissue ingrowth. Consistent with the micro-CT findings, the defect regions in the blank control and pure PCL NFs groups were mainly occupied by loose fibrous connective tissue at both 4 and 8 weeks, with limited new bone formation. In contrast, the 3D hierarchical scaffold groups exhibited more evident and progressive osteogenesis. At 4 weeks post-implantation, bone lacunae and central canal-like structures were observed within the defect sites treated with the CaSiO_3_/PCL NFs + PCL MSs and the NIR-irradiated CaSiO_3_/PCL NFs + BP@PCL MSs scaffolds (Fig. 10c). Meanwhile, neovascularization was also observed in the surrounding soft tissues, suggesting early angiogenic activity during the repair process.

To further evaluate vascularization within the defect region, the CD31-positive area was quantified using ImageJ (Fig. S16, Supporting Information). The quantitative results were generally consistent with the histological observations. Notably, relatively high CD31-positive areas were also observed in the Blank and PCL NFs groups. This may be attributed to the presence of vascularized fibrous connective tissue within the defect region rather than newly formed bone tissue. In contrast, in the CaSiO_3_/PCL NFs + BP@PCL MSs + NIR group, CD31-positive signals were accompanied by more evident new bone formation and collagen deposition, suggesting that angiogenesis in this group was more closely associated with the bone regeneration process.

By week 8, more mature mineralized bone tissue were observed in the CaSiO_3_-incorporated and BP/NIR-activated groups. Notably, the defect treated with the CaSiO_3_/PCL NFs + BP@PCL MSs scaffold under NIR irradiation showed substantial bony bridging and improved bone remodeling, with histological features approaching those of native bone. This favorable regenerative effect may be attributed to the combined regulation of angiogenesis and osteogenesis. The release of silicate-related species and mild NIR-mediated photothermal stimulation may facilitate vascular ingrowth into the scaffold interior, thereby supporting nutrient supply during tissue regeneration. Meanwhile, the sustained release of Ca^2+^ and PO_4_^3−^ contributes to the formation of a biomineralization-favorable microenvironment, thereby promoting new bone formation and structural remodeling. From an application perspective, this scaffold design may be particularly suitable as a locally implantable regenerative scaffold for non-load-bearing or low-load-bearing bone defects, such as craniofacial bone defects, where early structural guidance, bioactive ion regulation, and externally controllable photothermal stimulation are beneficial for promoting defect repair. This multifunctional and externally regulatable strategy may provide a useful design reference for future bone tissue engineering scaffolds with improved regenerative and translational potential.

## 4 Conclusion

In summary, we successfully developed a hierarchical 3D nanofibrous scaffold with integrated photothermal activity and ion-regulated mineralization capacity by combining electrospinning and coaxial electrospraying technologies. In this scaffold, CaSiO_3_/PCL nanofibers provided a structurally supportive and osteoinductive matrix, while BP@PCL microspheres acted as both a photothermal component and a controlled phosphate source. Under NIR irradiation, the scaffold exhibited effective antibacterial activity and promoted the regulated release of Ca^2+^, PO_4_^3−^, and silicate-related species, thereby establishing a favorable microenvironment for BMSC proliferation, osteogenic differentiation, and biomimetic mineralization. In vivo results further demonstrated that this multifunctional scaffold enhanced vascularized bone regeneration in cranial defect models. Therefore, the CaSiO_3_/PCL NFs + BP@PCL MSs scaffold may serve as a locally implantable regenerative material for non-load-bearing or low-load-bearing bone defects, such as craniofacial bone defects, alveolar ridge preservation, and periodontal bone defects. This membrane-like scaffold is mainly designed to maintain early-stage 3D structural support, regulate the local ionic microenvironment, and provide NIR-responsive antibacterial activity, thereby supporting vascularized bone regeneration. This photothermal–ionic dual-regulation strategy offers a useful design reference for future bioactive scaffolds in bone tissue engineering.

## CRediT authorship contribution statement

**Xinyue Guan:** Investigation, Methodology, Software, Validation, Visualization, Writing – original draft. **Siyu Xu:** Investigation, Methodology, Validation, Visualization, Writing – original draft. **Wenxin Meng:** Software, Validation, Visualization. **Quanli Li:** Formal analysis, Supervision. **Yuhui Liu:** Investigation. **Chuan Wu:** Validation. **Zhongrong Chen:** Conceptualization, Data curation, Supervision, Writing – review & editing. **Guomin Wu:** Conceptualization, Data curation, Project administration, Resources, Supervision, Writing – review & editing.

## Declaration of competing interest

The authors declare that they have no known competing financial interests or personal relationships that could have appeared to influence the work reported in this paper.

## Data Availability

Data will be made available on request.
