# Supplementary material for: Black phosphorus/calcium silicate-functionalized 3D hierarchical scaffolds: Coupling photothermal therapy with ion microenvironment-induced mineralization for bone repair
Source: Mater Today Bio. 2026 Jul 10;39:103446. doi: 10.1016/j.mtbio.2026.103446 (PMC13383259; doi:10.1016/j.mtbio.2026.103446)
Supplement: Multimedia component 1 [file mmc1.docx]

**Supporting Information**

**Black Phosphorus/Calcium Silicate-Functionalized 3D Hierarchical Scaffolds: Coupling Photothermal Therapy with Ion Microenvironment-Induced Mineralization for Bone Repair**

Xinyue Guan^a, 1^, Siyu Xu^b, 1^, Wenxin Meng^a, 1^, Quanli Li^a^, Yuhui Liu^a^, Chuan Wu^a^, Zhongrong Chen^b, *^, Guomin Wu^a, *^

*^a^* College & Hospital of Stomatology, Anhui Medical University, Key Lab. of Oral Diseases Research of Anhui Province, Hefei, 230032, China.

*^b^* School of Biomedical Engineering, Anhui Medical University, Hefei 230023, China.

^1^ These authors contributed equally to this work.

* Corresponding authors.

E-mail addresses: [zhrchen@ahmu.edu.cn](mailto:zhrchen@ahmu.edu.cn); [Wuguomin@ahmu.edu.cn](mailto:Wuguomin@ahmu.edu.cn);

**Supplementary Figures**

**
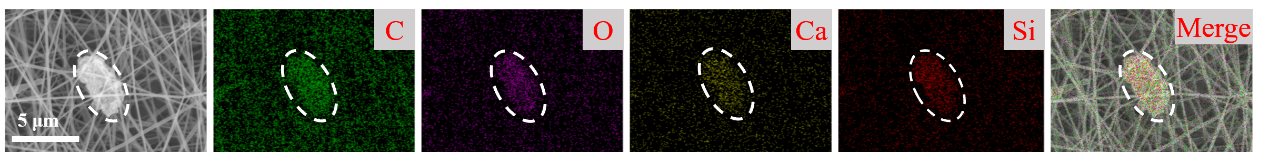
Fig. S1**. EDS elemental mappings of the CaSiO_3_/PCL NFs


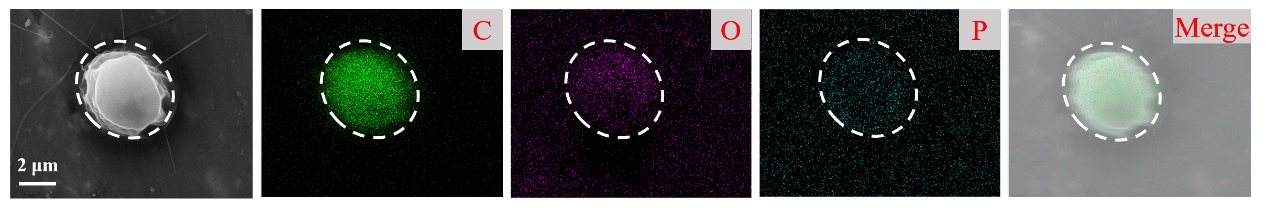
**Fig. S2.** EDS elemental mappings of the BP@PCL MSs

**Fig. S3.** SEM images of the PCL NFs and PCL NFs + PCL MSs cross sections.


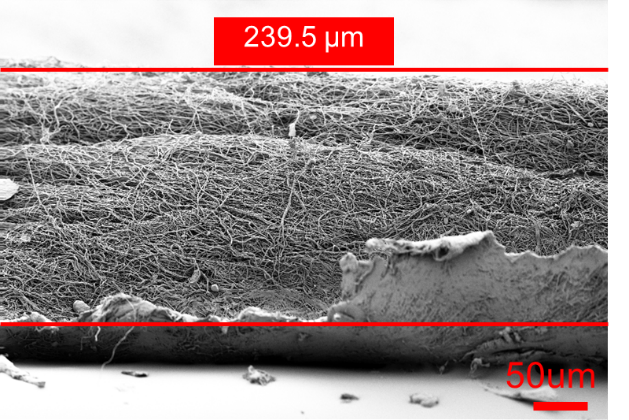

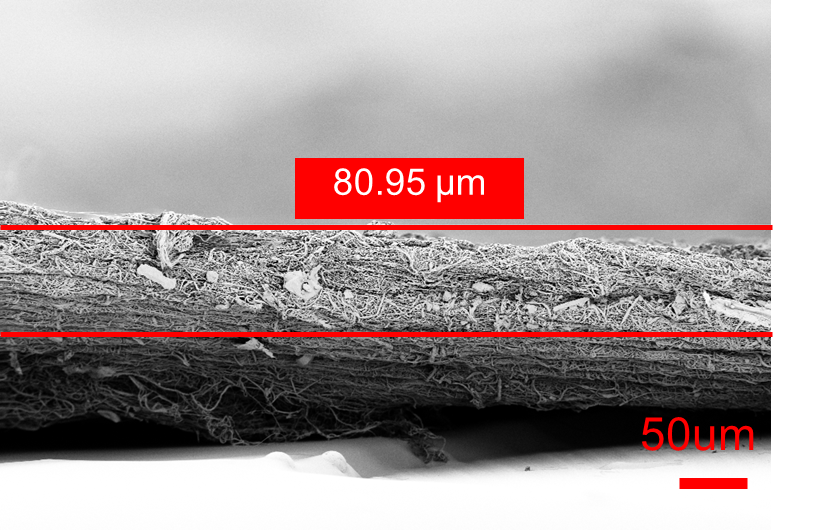


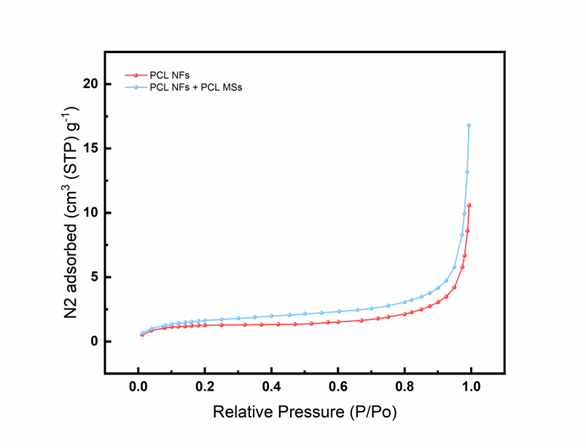
**Fig. S4.** Nitrogen adsorption-desorption isotherms of PCL NFs and PCL NFs + PCL MSs scaffolds.


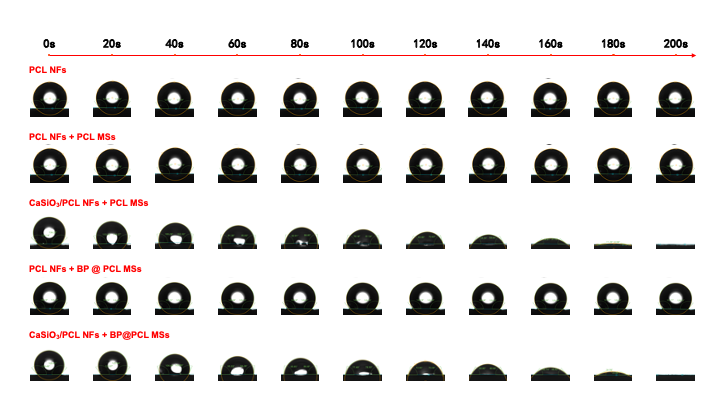
**Fig. S5.** Water contact angle observation of PCL NFs, PCL NFs + PCL MSs, CaSiO_3_/PCL NFs + PCL MSs, PCL NFs + BP @ PCL MSs and CaSiO_3_/PCL NFs + BP@PCL MSs scaffolds.


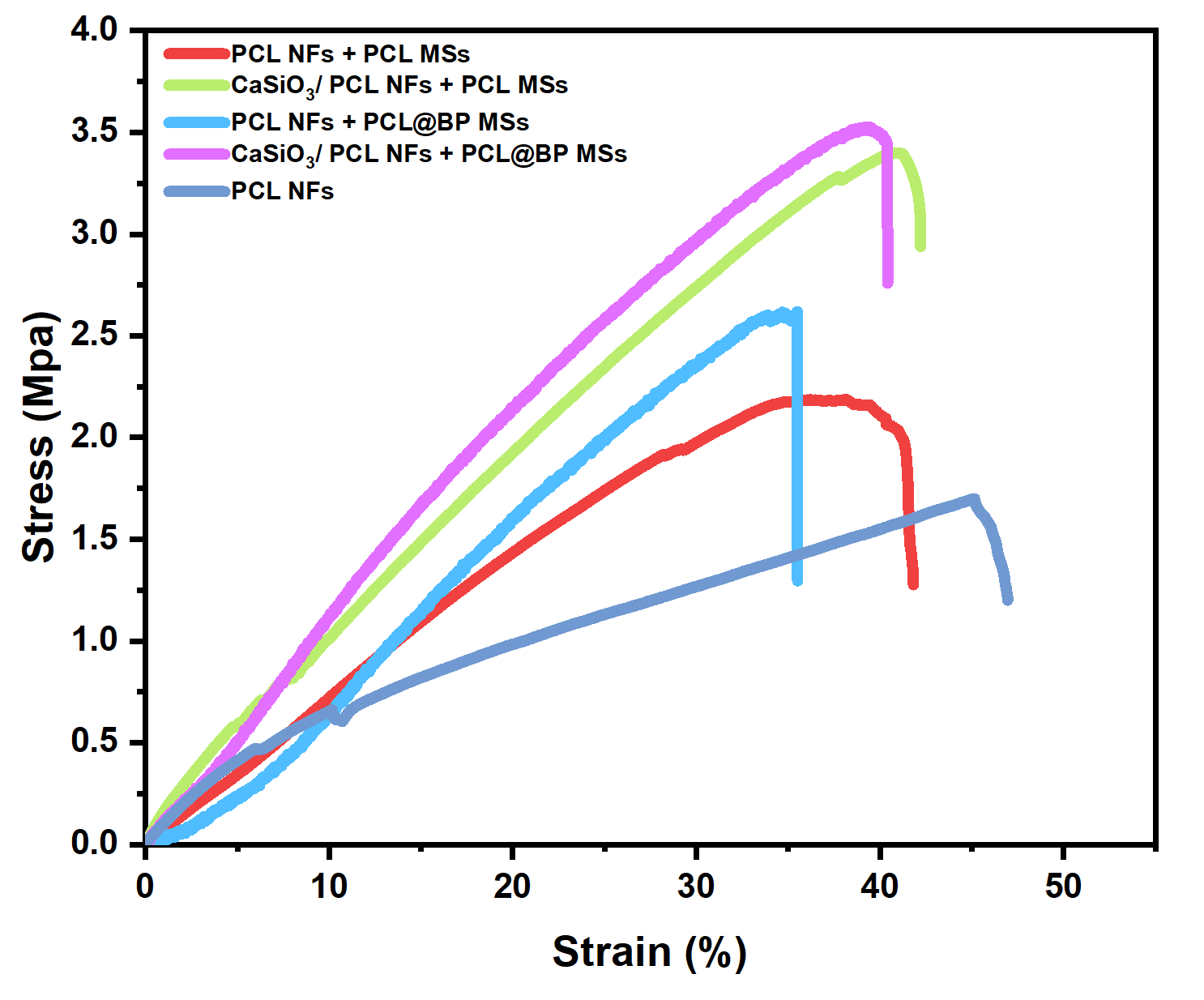
**Fig. S6.** Stress–strain curves of PCL NFs, PCL NFs + PCL MSs, CaSiO_3_/PCL NFs + PCL MSs, PCL NFs + BP @ PCL MSs and CaSiO_3_/PCL NFs + BP@PCL MSs scaffolds.





**Fig. S7.** The temperature of CaSiO_3_/PCL NFs + BP@PCL MSs scaffold under NIR exposure with different powers.


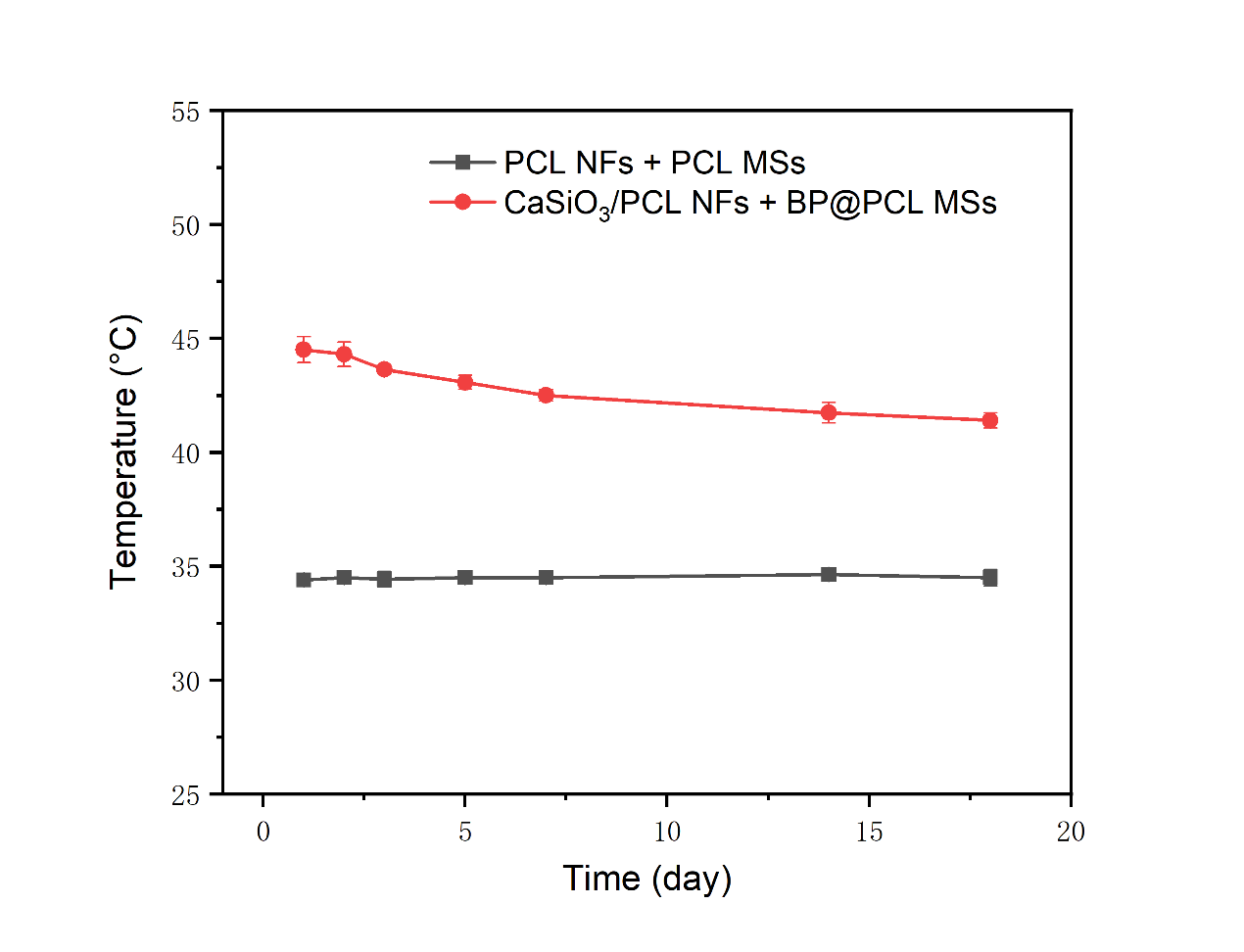
**Fig. S8**. Temperature curve of the internal scaffold in the rat skull after NIR.


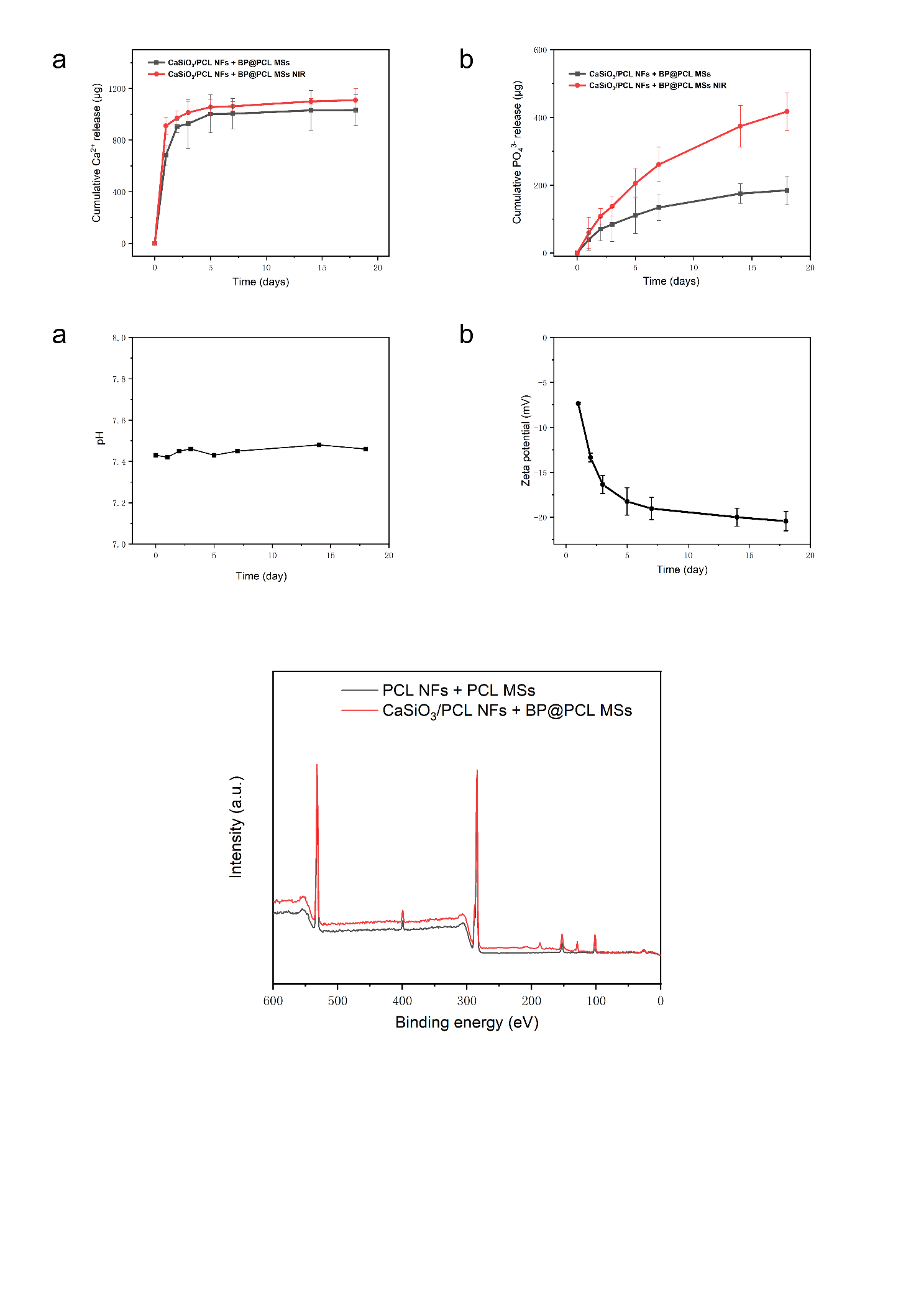
**Fig. S9**. a) Ca²⁺ release curves; b) PO₄³⁻ release curves for scaffolds CaSiO₃/PCL NFs + BP@PCL MSs (with and without NIR)


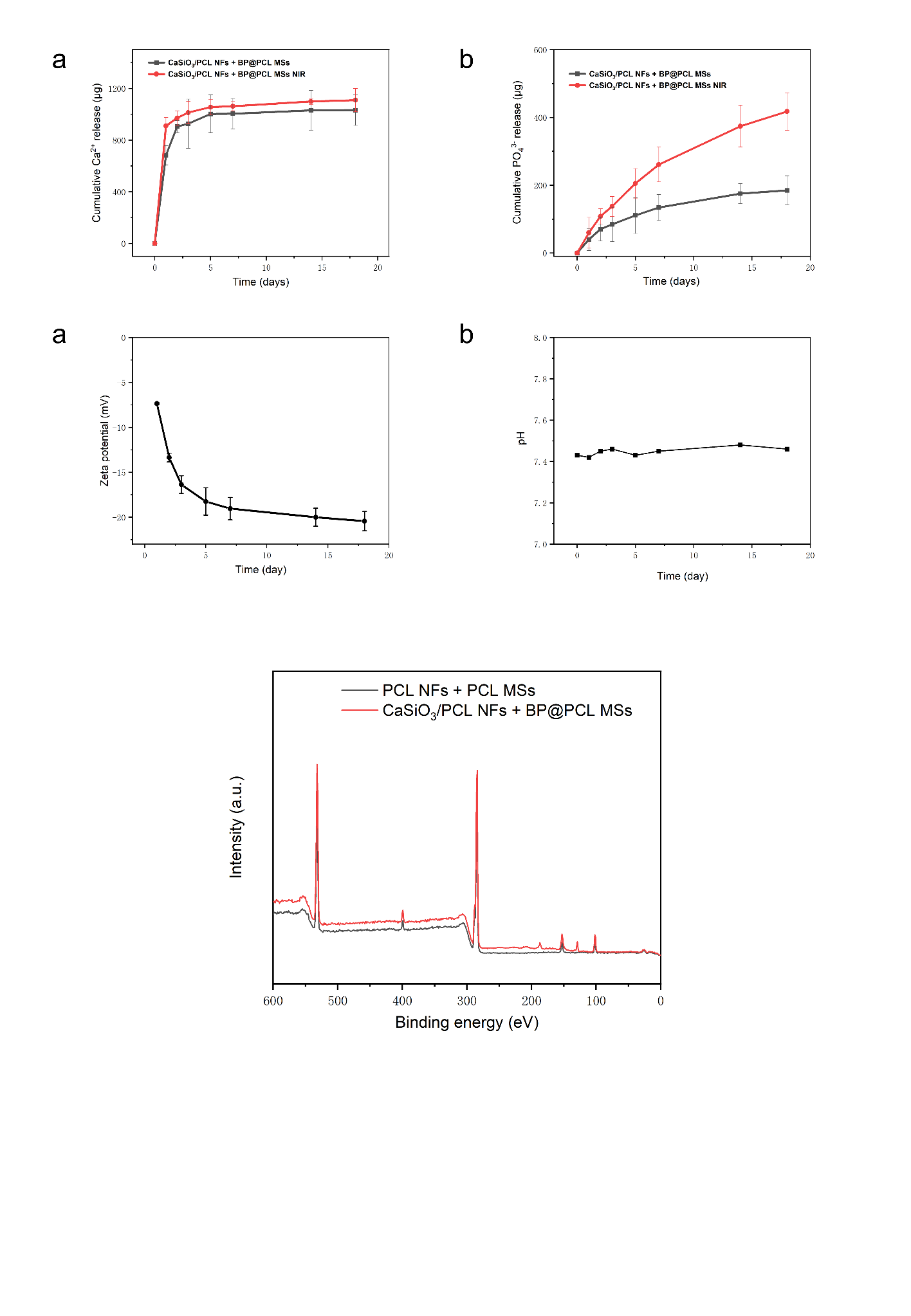
**Fig. S10**. a) zeta value curve; b) pH curve of the PBS solution used to soak the CaSiO₃/PCL NFs + BP@PCL MSs scaffold during the degradation process
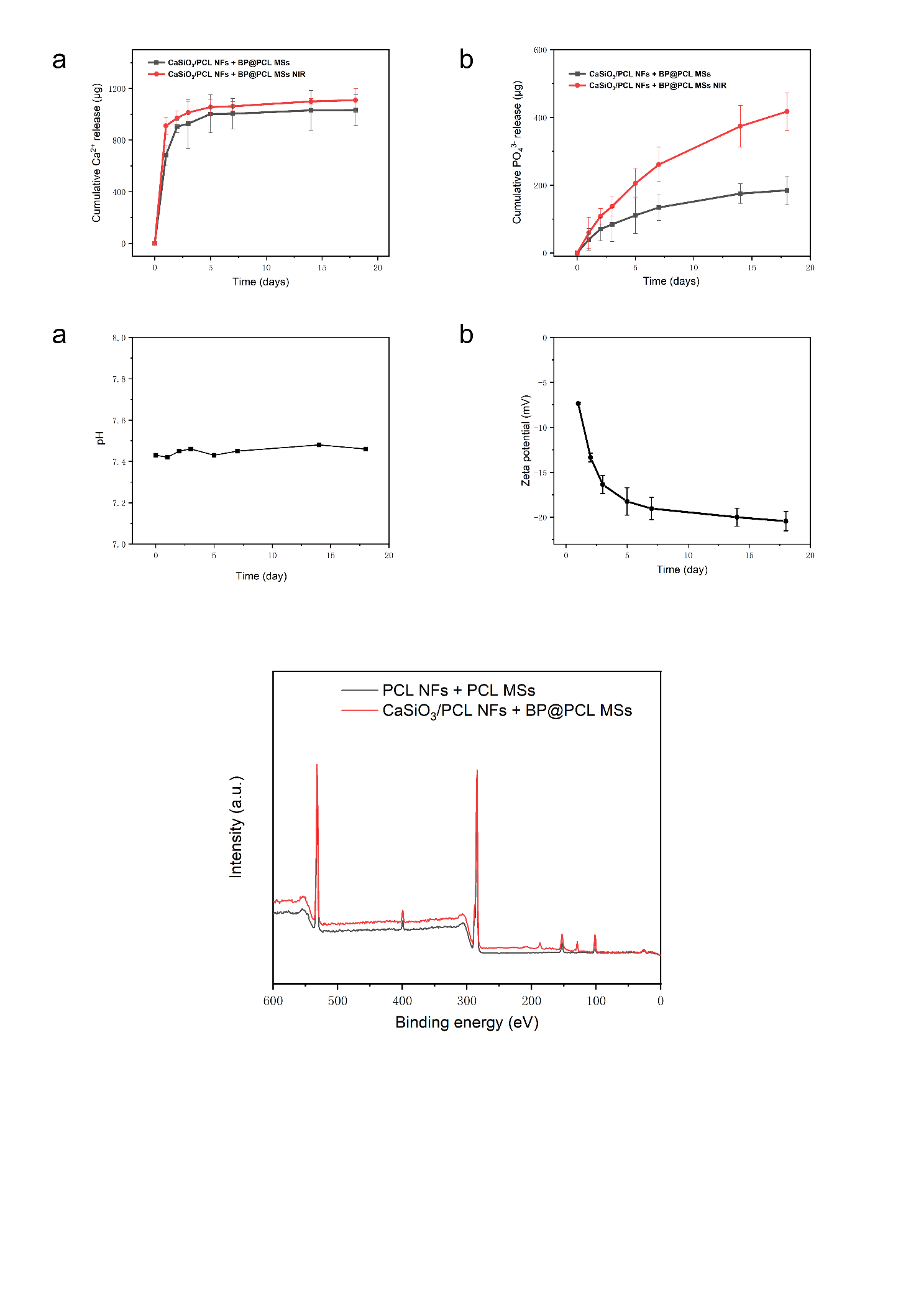


**Fig. S11**.a) Ca²⁺ release curves; b) PO₄³⁻ release curves for scaffolds CaSiO₃/PCL NFs + BP@PCL MSs (with and without NIR)

**
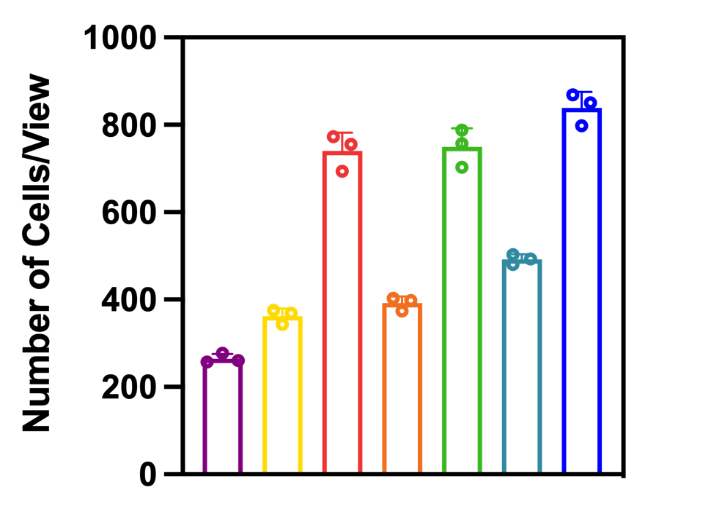
**

**Fig. S12.** Numbers of cells on the different groups of scaffolds after 3 day of BMSC cells culture (n = 3)


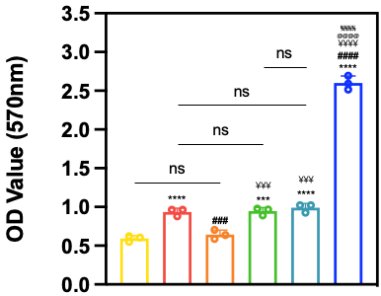

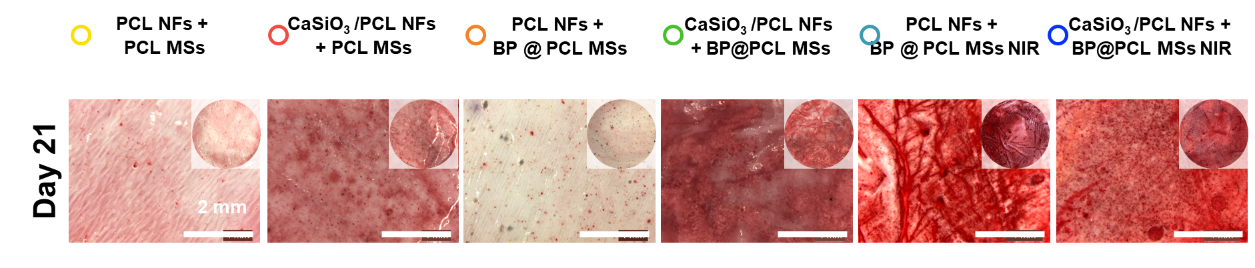


**Figure S13.** Alizarin red staining and semi-quantification of osteogenic-induced cells on the scaffold surface after 21 days.


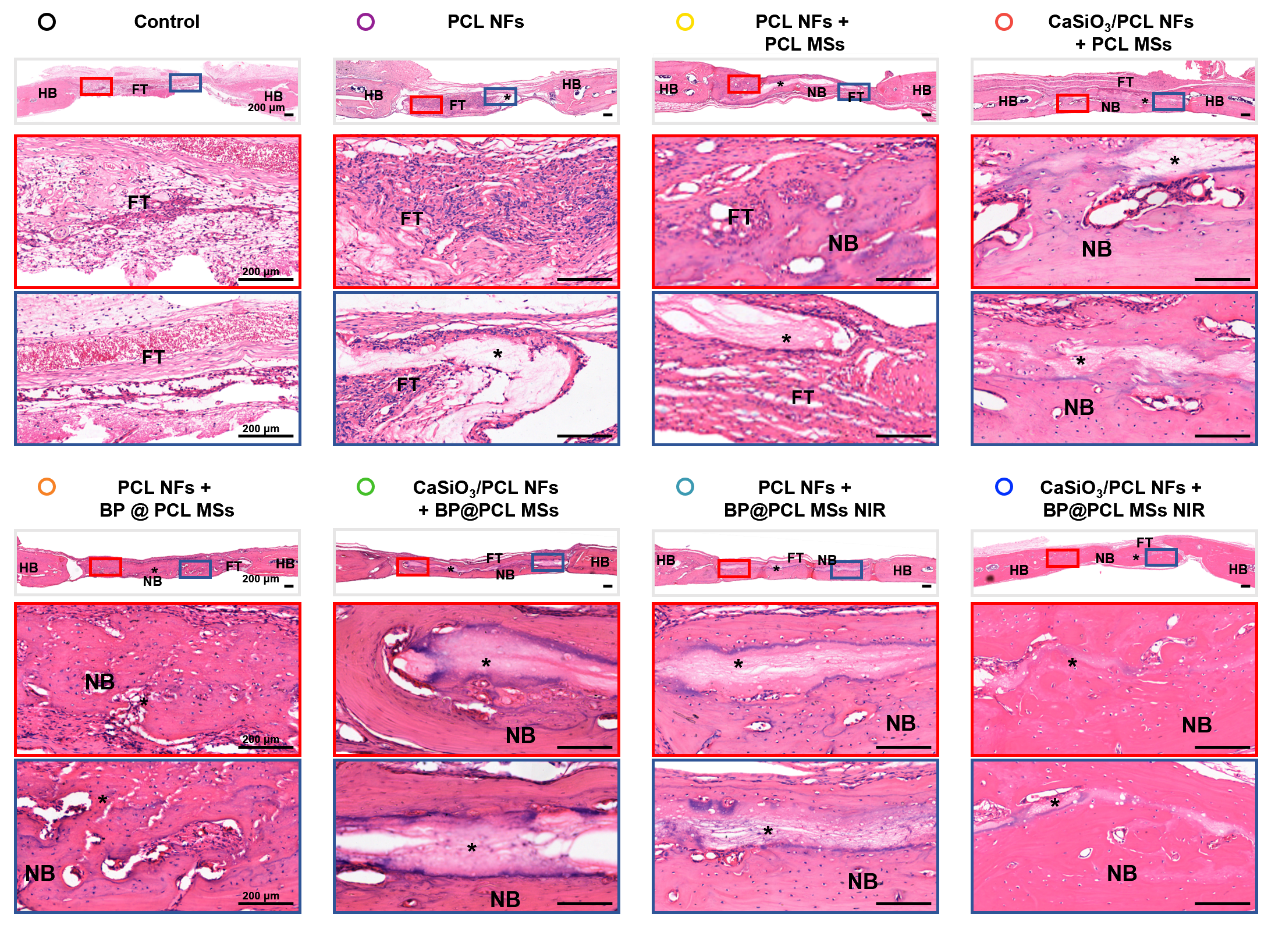


**Fig. S14.** HE staining of cranial defects at 8 weeks post-implantation.


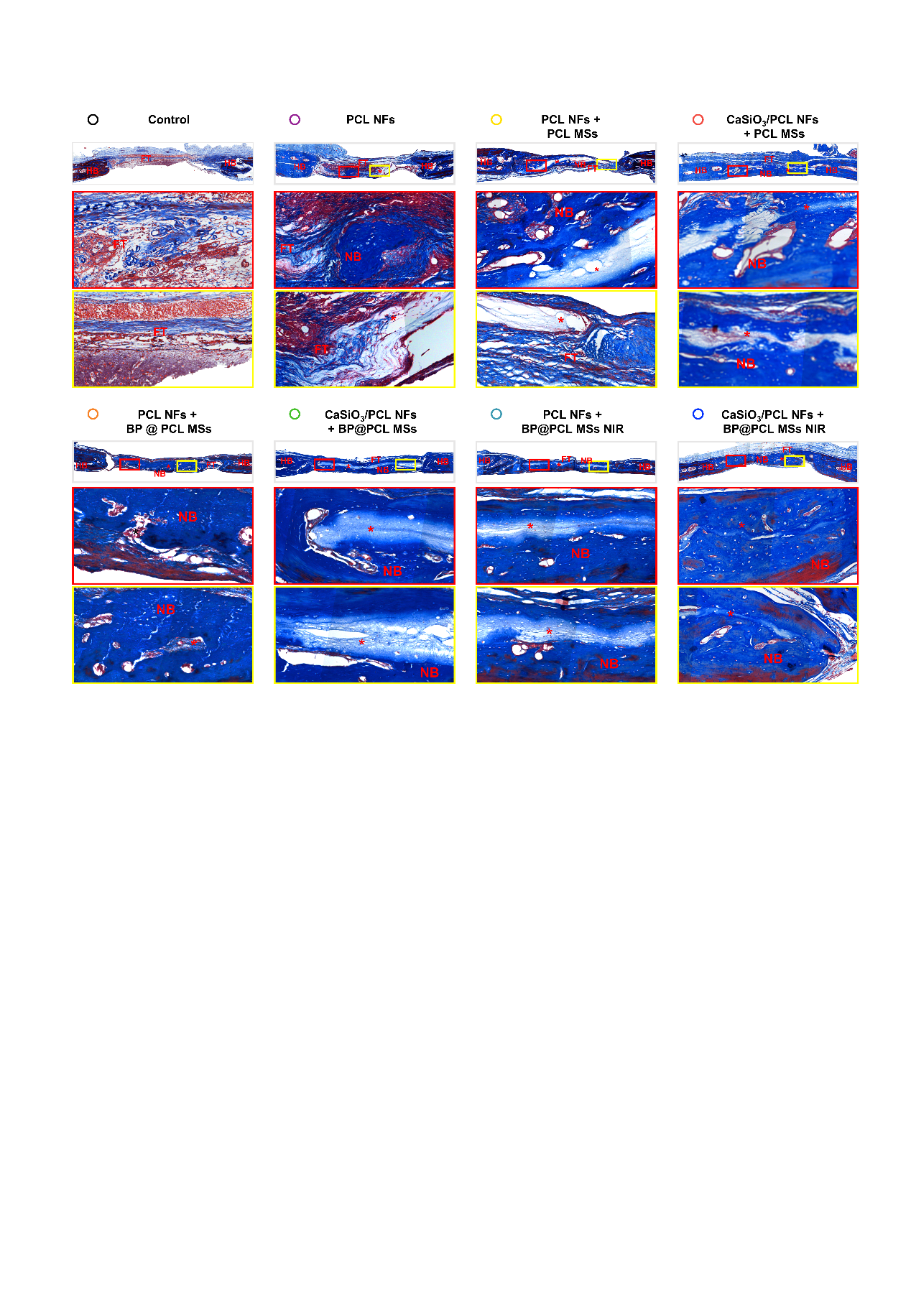
 **Fig. S15.** Masson staining of cranial defects at 8 weeks post-implantation.


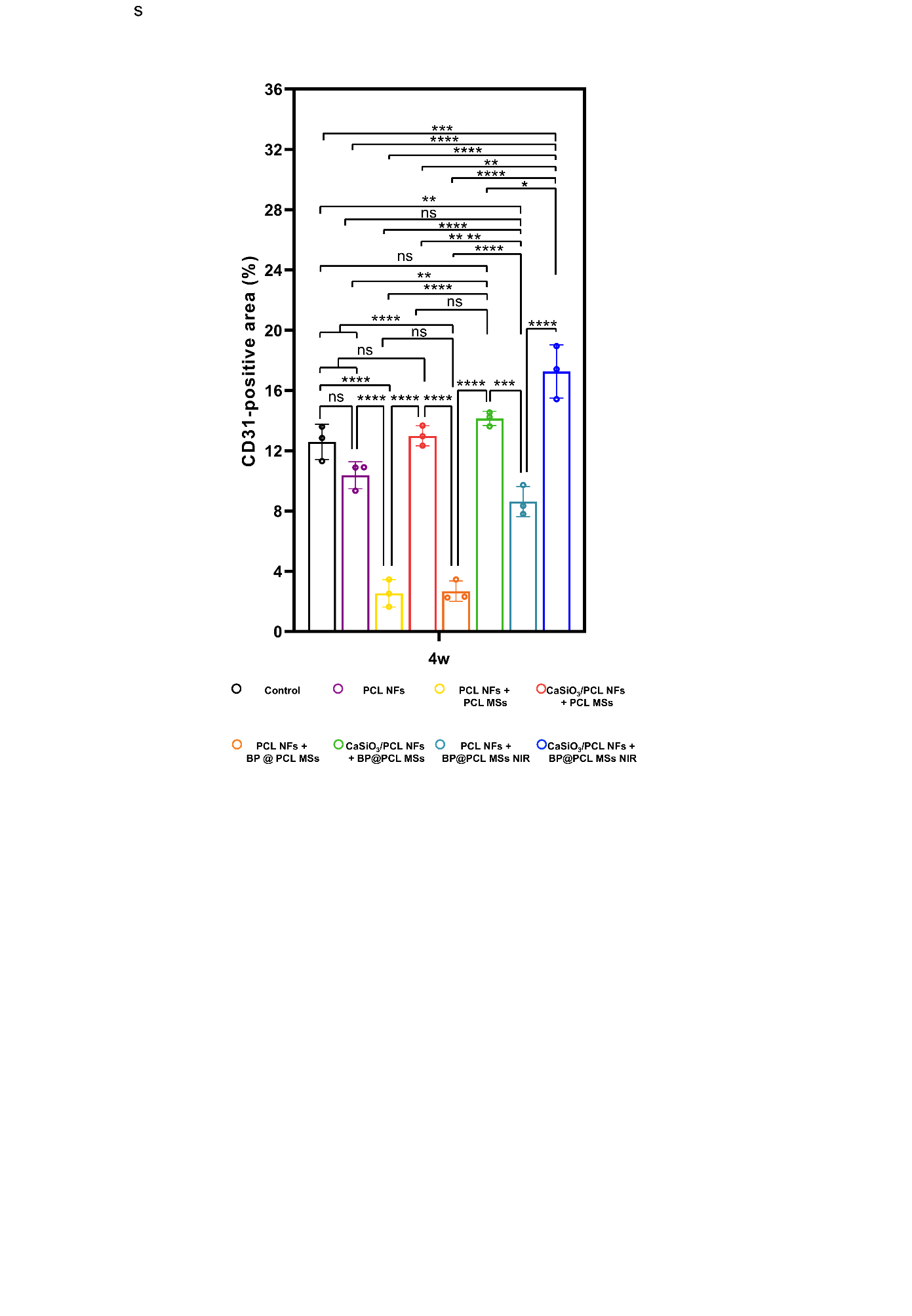


**Fig. S16.** Masson staining of cranial defects at 8 weeks post-implantation.
